# Designing a novel multi‑epitope vaccine against Ebola virus using reverse vaccinology approach

**DOI:** 10.1038/s41598-022-11851-z

**Published:** 2022-05-11

**Authors:** Morteza Alizadeh, Hossein Amini-Khoei, Shahram Tahmasebian, Mahdi Ghatrehsamani, Keihan Ghatreh Samani, Yadolah Edalatpanah, Susan Rostampur, Majid Salehi, Maryam Ghasemi-Dehnoo, Fatemeh Azadegan-Dehkordi, Samira Sanami, Nader Bagheri

**Affiliations:** 1 Department of Tissue Engineering, School of Medicine, Shahroud University of Medical Sciences, Shahroud, Iran; 2 Medical Plants Research Center, Basic Health Sciences Institute, Shahrekord University of Medical Sciences, Shahrekord, Iran; 3 Department of Medical Biotechnology, School of Advanced Technologies, Shahrekord University of Medical Sciences, Shahrekord, Iran; 4 Department of Microbiology and Immunology, Faculty of Medicine, Shahrekord University of Medical Sciences, Shahrekord, Iran; 5 Clinical Biochemistry Research Center, Basic Health Sciences Institute, Shahrekord University of Medical Sciences, Shahrekord, Iran; 6 Cellular and Molecular Research Center, Yasuj University of Medical Sciences, Yasuj, Iran; 7 Department of Molecular Medicine, School of Advanced Medical Science and Technology, Shiraz University of Medical Science, Shiraz, Iran

**Keywords:** Biotechnology, Computational biology and bioinformatics

## Abstract

Ebola virus (EBOV) is a dangerous zoonotic infectious disease. To date, more than 25 EBOV outbreaks have been documented, the majority of which have occurred in Central Africa. The rVSVG-ZEBOV-GP vaccine (ERVEBO), a live attenuated vaccine, has been approved by the US Food and Drug Administration (FDA) to combat EBOV. Because of the several drawbacks of live attenuated vaccines, multi-epitope vaccines probably appear to be safer than live attenuated vaccines. In this work, we employed immunoinformatics tools to design a multi-epitope vaccine against EBOV. We collected sequences of VP35, VP24, VP30, VP40, GP, and NP proteins from the NCBI database. T-cell and linear B-cell epitopes from target proteins were identified and tested for antigenicity, toxicity, allergenicity, and conservancy. The selected epitopes were then linked together in the vaccine's primary structure using appropriate linkers, and the 50S ribosomal L7/L12 (Locus RL7 MYCTU) sequence was added as an adjuvant to the vaccine construct's N-terminal. The physicochemical, antigenicity, and allergenicity parameters of the vaccine were all found to be satisfactory. The 3D model of the vaccine was predicted, refined, and validated. The vaccine construct had a stable and strong interaction with toll-like receptor 4 (TLR4) based on molecular docking and molecular dynamic simulation (MD) analysis. The results of codon optimization and in silico cloning revealed that the proposed vaccine was highly expressed in *Escherichia coli* (*E. coli*). The findings of this study are promising; however, experimental validations should be carried out to confirm these findings.

## Introduction

Ebola virus (EBOV) is a hazardous zoonotic infectious disease that was first identified in 1976 when it spread simultaneously in South Sudan and a village near the Ebola River in Yambuku city of Democratic Republic of Congo^1,2^. Since then, more than 25 EBOV outbreaks have been reported, the majority of which have occurred in Central Africa^1^. The 2014–2016 outbreak in West Africa was the largest documented outbreak of EBOV, which infected over 28,000 people and caused over 11,000 deaths^3^. The African continent's favorable environmental conditions help in promoting EBOV endemicity. At least one human case of one or more Ebolavirus species has been reported in thirteen African countries^4^. Regrettably, intermittent imported EBOV cases have been reported in the United States, United Kingdom, Thailand, Spain, and Canada^5,6^.

EBOV is transmitted from wild animals to humans and spreads through human-to-human transmission^7^. Human-to-human transmission of EBOV occurs through direct contact with infected people's skin, contact with blood and body fluids, and sexual intercourse, handling, secretions, organs, or other bodily fluids of infected people^8^. The symptoms of EBOV disease, formerly known as EBOV hemorrhagic fever, include fever, headache, fatigue, muscle aches, internal and external bleeding, diarrhea, vomiting, and impaired kidney and liver function^5,9,10^.

The genus Ebolavirus belongs to the *Filoviridae* family and includes six species: Bombali ebolavirus, Bundibugyo ebolavirus, Reston ebolavirus, Sudan ebolavirus, Tai Forest ebolavirus, and Zaire ebolavirus^11^. The EBOV are viruses that contain linear, non-segmented, negative-sense, single-stranded genomic RNA^12^. Replication promoters are encoded at the 3- and 5-ends of the 19-kb genome, which are known as the 3-leader and 5-trailer, respectively^13^. The EBOV genome contains seven genes coding for nine mRNAs and proteins, including the nucleoprotein (NP), viral RNA polymerase cofactor (VP35), matrix proteins (VP40), spike glycoprotein (GP) (3 variants), transcriptional activator (VP30), second matrix protein (VP24), and RNA polymerase (L)^14^. The NP is responsible for genome encapsulation during virus assembly and the protection of viral RNA from degradation^15^. The VP35 interacts with NP and L during the EBOV RNA replication process, and both VP35-NP and VP35-L interactions are required for viral RNA synthesis^16,17^. The role of VP40 is vital in the final stage of EBOV replication, when viral particles bud from host cells^18–20^. VP40 attaches the viral nucleocapsid to the inside of the cell membrane and facilitates egress to complete the viral life cycle^21^. The GP is responsible for EBOV attachment and entry into host cells, as well as inducing immune responses in host cells^22,23^. The VP30 is a phosphorylation-dependent viral transcription factor that is required to initiate RNA transcription^24^. The minor viral matrix protein VP24 condenses viral nucleocapsids, which is necessary for efficient nucleocapsid packaging into the virion^25–27^. The L protein is an RNA-dependent polymerase that complexes with VP30 and performs all of the enzymatic activities that are required for virus genome transcription and replication^28^.

**Figure 1:**
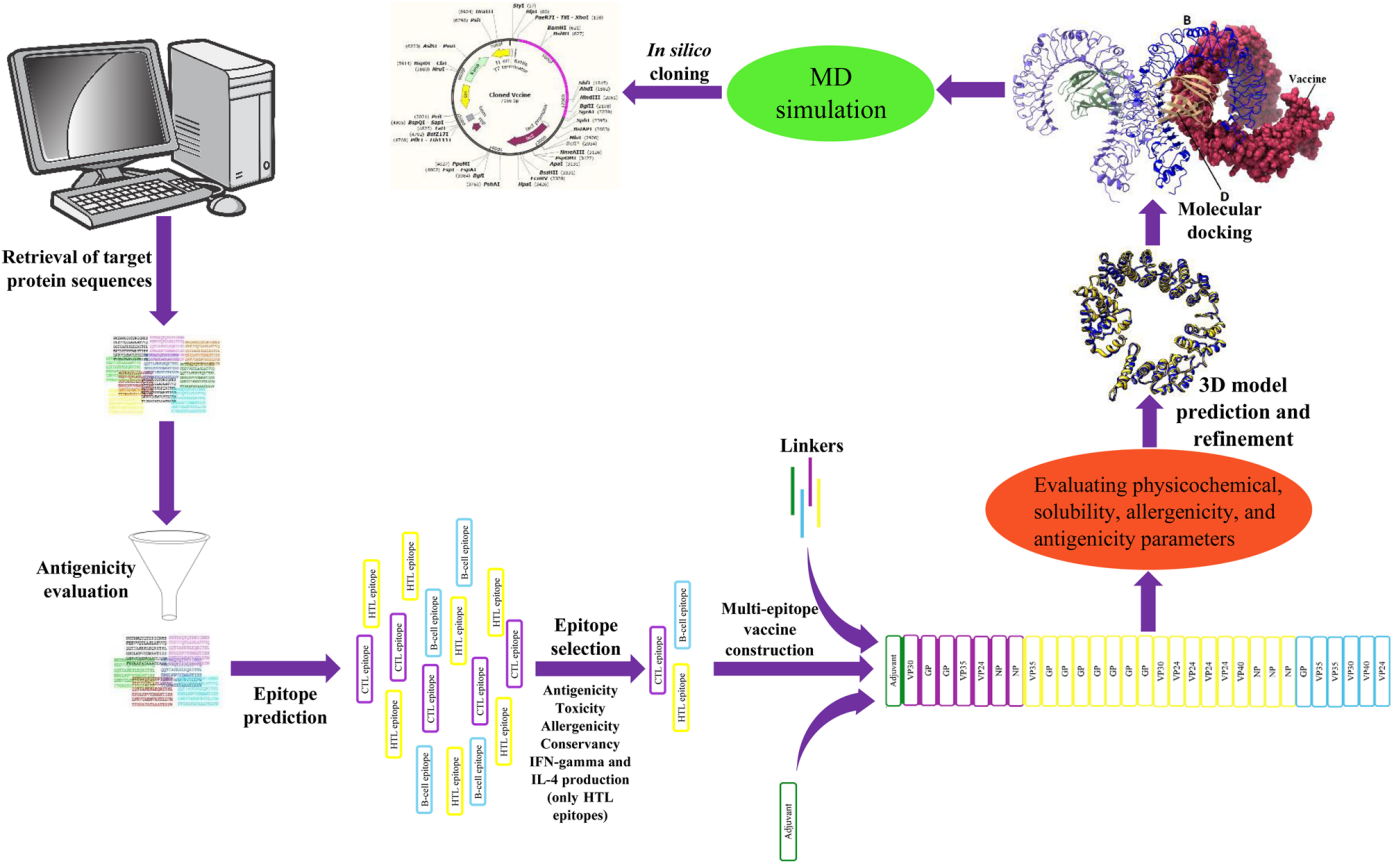
A schematic workflow of constructing a multi-epitope vaccine against EBOV.

Only a few therapeutics for EBOV disease have been developed and tested to now. The US Food and Drug Administration (FDA) has approved two EBOV-specific monoclonal antibody therapies, REGN-EB3 and mAb114, for the treatment of EBOV disease^29^. As mentioned, various African countries, the majority of which are low- and middle-income, are more susceptible to this disease. Therefore, more affordable treatments that are easily stored and useful for low- and middle-income countries are required^30^. Vaccines are critical for managing infectious diseases, particularly among people living in poor health. Vaccines can also have herding effects, resulting in protection even among individuals who have not been vaccinated, which is especially important for the poor who lack access to health care^31^. The rVSVΔG-ZEBOV-GP vaccine (ERVEBO) was approved on December 19, 2019, by the FDA to prevent EBOV infection in people 18 years of age and older^32^. The ERVEBO is a live attenuated vaccine that was created by replacing the gene encoding the glycoprotein of the vesicular stomatitis virus strain Indiana with the gene encoding the glycoprotein of the EBOV-Kikwit 1995 strain^33^. Due to numerous disadvantages of live attenuated vaccines, such as the possibility of reversion to virulent form and instability in various storage conditions, it appears that multi-epitope vaccines appear to be safer than live attenuated vaccines^34^. The multi-epitope vaccines have received a lot of attention because they have higher immunity, lower allergic reactions, and easier production than conventional vaccines^35^. The main downside of these vaccines is their low immunogenicity, which is overcome by the use of adjuvants^36^. The majority of in silico studies on the design of an Ebola vaccine has progressed to the point of predicting epitopes. The vaccine construct has been designed in several studies, although bioinformatics analyses on it have not been extensive. The aim of this study is to in silico design a vaccine construct for the first time that includes the epitopes of VP35, VP24, VP30, VP40, GP, and NP proteins, as well as a 50S ribosomal L7/L12 sequence as an adjuvant. In this regard, target proteins were first selected to predict T-cell and B-cell epitopes. The epitopes were then screened based on their antigenicity, toxicity, allergenicity, conservancy, and potential to produce IFN-gamma and IL-4. The top-ranked epitopes were arranged in a vaccine construct using appropriate linkers. To improve vaccine immunogenicity, the 50S ribosomal L7/L12 (Locus RL7 MYCTU) sequence (as a TLR4 agonist) was added to the vaccine construct's N-terminal as an adjuvant. Through the TLR4, 50S ribosomal L7/L12 can induce immature dendritic cells and naive T cells and elicit an adaptive immune response^37^. The vaccine construct was evaluated for physicochemical characterisation, solubility, allergenicity, and antigenicity. The secondary structure and 3D structure of the vaccine construct were modeled, and the 3D model was refined. Further, molecular docking of the 3D model with TLR4 was performed, subsequently, the docked complex was subjected to molecular dynamics (MD) simulation to analyze its structural dynamics and stability. Finally, codon optimization and in silico cloning were done to confirm the vaccine's effective expression. Figure 1 depicts the step-by-step workflow employed in this study.

## Results

### Identification and retrieval of viral protein sequences

Based on previous studies^38–40^ and the Protegen server, the VP35, VP24, VP30, VP40, GP, and NP proteins were chosen as target proteins for epitope prediction. The VaxiJen v2.0 server predicted that all target proteins had an antigen score of > 0.4 (threshold value); their accession number and antigenicity scores are given in Table 1.

**Table 1:** Details of the proteins used in the design of the vaccine.

Protein	Accession number	VaxiJen score
VP35	NP_066244.1	0.5129
VP24	NP_066250.1	0.4735
VP30	NP_066249.1	0.5221
VP40	NP_066245.1	0.5103
GP	NP_066246.1	0.4946
NP	NP_066243.1	0.4468

### T-cell epitope prediction

Cytotoxic T lymphocytes (CTLs) are critical elements of adaptive immunity that play a key role in the elimination of infected cells^41^. CTL epitopes are involved in the development of long-lasting cellular immunity and can remove circulating viruses as well as virus-infected cells^42^. The NetCTL 1.2 server identified a total of 591 CTL epitopes from six proteins. To determine the best CTL epitopes from a set of CTL epitopes, we first chose CTL epitopes that bind strongly to at least three MHC class I supertypes. These epitopes were then filtered for antigenicity, toxicity, allergenicity, and conservancy. Finally, ten antigenic, non-toxic, and non-allergenic CTL epitopes with more than 80% conservancy were chosen (Tables S1–S6).

Helper T lymphocytes (HTLs) play an important in the elimination of extracellular pathogens using various cytokines and stimulating humoral immune responses^43^. HTL epitopes help regulate the adaptive immune system by inducing the production of T cell cytokines^44^. For the six target proteins, 912 HTL epitopes of 15-mer length were predicted by the NetMHCII 2.3 server. Among them, HTL epitopes that could bind strongly to at least 5 human MHC class II alleles were screened for antigenicity, toxicity, allergenicity, and conservancy, as well as the production of cytokines such as IFN-gamma and IL-4. Considering the mentioned characteristics, a total of 17 HTL epitopes were selected (Tables S7–S12).

### Linear B-cell epitope prediction

B-cells are the main body of humoral immunity. Antibodies produced by B-cells play a critical role in preventing the spread of viral infections^45^. Linear B cell epitopes are responsible for producing antigen-specific antibodies^46^. We predicted 68 linear B-cell epitopes using the IEDB server in this study (Fig. S1). The epitopes with a size of 10–30 mer were tested for antigenicity, toxicity, allergenicity, and conservancy. Finally, seven epitopes were chosen for the VP35, VP24, VP30, VP40, and GP proteins, while no epitopes were chosen for the NP protein (Tables S13–S18).

### Multi-epitope vaccine construction

**Figure 2:**
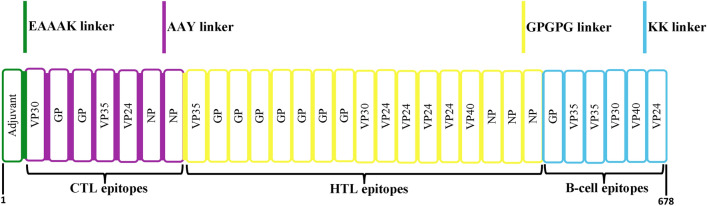
A schematic depiction of the final multi-epitope vaccine construct.

To avoid epitopes repetition in the vaccine construct, epitopes whose sequences were found in other epitopes were eliminated. A total of 7 CTL, 17 HTL, and 6 linear B-cell epitopes were fused using AAY, GPGPG, and KK linkers, respectively (Table 2). In addition, the 50S ribosomal L7/L12 adjuvant (with a length of 130 amino acids) was attached to the N-terminal of the vaccine sequence by an EAAAK linker. The final vaccine structure included 678 amino acids (Fig. 2).

**Table 2:** A list of the epitopes that constitute the multi-epitope vaccine.

Protein	CTL epitopes	HTL epitopes	Linear B-cell epitopes
VP35	FQLQDGKTL	CALIQITKRVPIFQD	QQTIASESLEQRITSLEN RGDIPRACQKSLRPVPPSPKID
VP24	RMQSLILEF	EQLSLKMLSLIRSNI NHFNMRTQRVKEQLS NTNHFNMRTQRVKEQ TNHFNMRTQRVKEQL	KTNDFAPAWSM
VP30	ITAFLNIAL	LLTLCAVMTRKFSKS	PQSDNEEASTNPGTCSWSD
VP40	-	STTAAIMLASYTITH	LPNKSGKKGNSADLTSPE
GP	AIGLAWIPY GTNETEYLF	DRFKRTSFFLWVIIL EYLFEVDNLTYVQLE ILFQRTFSIPLGVIH LFEVDNLTYVQLESR NETEYLFEVDNLTYV RDRFKRTSFFLWVII RFKRTSFFLWVIILF	TLQVSDVDKLVCRDKLSSTNQL
NP	FPQLSAIAL SSLAKHGEY	QQGIVRQRVIPVYQV VQQGIVRQRVIPVYQ IKRTLAAMPEEETTE	–

### Evaluating physicochemical, solubility, allergenicity, and antigenicity parameters of the designed vaccine

Several important physicochemical features of the multi-epitope vaccine were characterized by the ExPASy ProtParam tool. The theoretical pI and GRAVY of the vaccine construct were estimated to be 9.09 and − 0.264, respectively. The half-life of the vaccine was calculated to be 30 h in mammalian reticulocytes (in vitro), more than 20 h in yeast (in vivo), and more than 10 h in *E. coli* (in vivo). The molecular weight was computed to be 73.15 kDa. For the vaccine construct, an aliphatic index of 81.47 and an instability index of 26.72 were predicted. The SOLpro server predicted that the multi-epitope vaccine was soluble with a probability of 0.819255. The AllerTOP v. 2.0 server predicted that the vaccine construct was non-allergen. The antigenicity of the vaccine sequence was predicted to be 0.958093 by ANTIGENpro and 0.5179 by the VaxiJen 2.0 server with a virus model and 0.7422 with a bacteria model at a threshold of 0.4.

### Secondary structure prediction

The secondary structure of the multi-epitope vaccine was generated by PDBsum. The vaccine construct was composed of 41 helices, 71 helix-helix interactions, 125 beta turns, and 22 gamma turns (Fig. S2).

### Tertiary structure prediction, refinement, and validation of the vaccine construct

**Figure 3:**
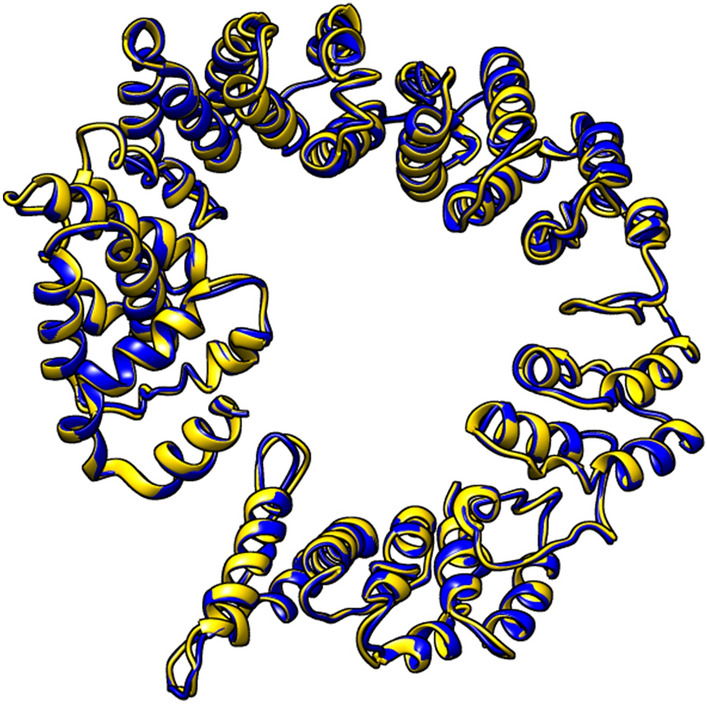
The initial 3D model of the vaccine is shown in blue and the refined 3D model is shown in gold. The UCSF Chimera 1.15rc software was used to visualize them.

I-TASSER server generated five 3D structures of the vaccine candidate based on threading templates (PDB Hit: 1rquA, 5lqwQ, 2ftc, 7ey7S, 7eybI, 1dd4, 7f56A, and 4uicA). According to the Z-score values (ranging from 1.26 to 4.74), all of the threading templates were good aligned, the Z-score > 1 indicates that the alignment is good, and vice versa^47^. Models 1–5 had C-scores of − 0.61, − 2.29, − 2.74, − 2.98, and − 4.36, respectively. For the refining process, the model with the highest C value (− 0.61) was chosen. The GalaxyRefne server was used to refine the selected model. The GalaxyRefne server provided a total of five refined models of the vaccine. Model 3 was considered the best refined model based on its model quality scores (Table 3). Higher GDT-HA values indicate higher quality models^48^; model 3 has a GDT-HA score of 0.9421, which was higher than all refined models. A lower RMSD value means more stability, and an RMSD score of 0 to 1.2 is normally considered acceptable^48^. The RMSD score for this model is 0.442. The MolProbity score represents the crystallographic resolution of the protein's 3D model. A lower MolProbity score indicates a less critical error^49^. Model 3 has a MolProbity score of 2.428, which is significantly lower than the initial model. The Clash score indicates the number of undesirable all-atom spatial overlaps, and the poor rotamers score indicates the number of residues with a lower capacity of conceivable rotation in their side chains^50^; the lower the score of these parameters, the better the 3D structure of the protein; the Clash and poor rotamers scores of model 3 are 23.9 and 0.9, respectively. The higher the Rama favored value, the higher the model's quality. Model 3 has Rama favored of 89.6. The initial and refined 3D model of the vaccine has been shown in Fig. 3. The quality of the initial and refined models of the multi-epitope vaccine was evaluated using the SWISS-MODEL Structure Assessment and ProSA-web server. Ramachandran plot analysis of the initial model revealed that 72.29% of the residues were in the favoured region, whereas in the refined model 88.76% of the residues were in the favoured region (Fig. S3). The z-scores calculated by the ProSA web server for the initial model and the refined model of the vaccine were − 2.46 and − 3.47, respectively (Fig. S3).

**Table 3:** Results of the GalaxyRefne server. Model 3 was selected as the best refined model based on the GDT-HA, RMSD, MolProbity, Clash score, Poor rotamers, and Rama favored parameters.

Model	GDT-HA	RMSD	MolProbity	Clash score	Poor rotamers	Rama favored
Initial	1.0000	0.000	3.291	25.1	17.4	72.2
Model 1	0.9381	0.455	2.593	24.9	1.5	88.8
Model 2	0.9411	0.451	2.436	24.2	1.1	89.2
Model 3	0.9421	0.442	2.428	23.9	0.9	89.6
Model 4	0.9373	0.448	2.526	24.5	1.5	89.3
Model 5	0.9397	0.454	2.595	24.1	1.7	89.6

### Discontinuous B-cell epitope prediction

The ElliPro server predicted 16 discontinuous B-cell epitopes with scores ranging from 0.585 to 0.829 (at a threshold of 0.5). The size of the epitopes varied from 8 to 39 residues (Table 4). Discontinuous B-cell epitopes with a score above 0.8 are shown in (Fig. S4).

**Table 4:** Predicted discontinuous B-cell epitopes from the refined 3D model of the multi-epitope vaccine.

No.	Start	End	Discontinuous B-cell epitopes	Number of residues	Score
1	618	656	RITSLENKKPQSDNEEASTNPGTCSWSDKKLPNKSGKKG	39	0.829
2	663	678	SPEKKKTNDFAPAWSM	16	0.812
3	463	475	NHFNMRTQRVKEQ	13	0.783
4	553	576	ETTEKKTLQVSDVDKLVCRDKLSS	24	0.737
5	91	111	KEAKDLVDGAPKPLLEKVAKE	21	0.729
6	52	74	AAVEAAEEQSEFDVILEAAGDKK	23	0.714
7	400	425	PGEQLSLKMLSLIRSNIGPGPGNHFN	26	0.715
8	161	180	TNETEYLFAAYFQLQDGKTL	20	0.708
9	360	385	PGRFKRTSFFLWVIILFGPGPGLLTL	26	0.709
10	521	542	GVQQGIVRQRVIPVYQGPGPGI	22	0.702
11	590	605	CQKSLRPVPPSPKIDK	16	0.701
12	137	144	TAFLNIAL	8	0.671
13	430	450	RVKEQLSGPGPGNTNHFNMRT	21	0.669
14	194	217	AYFPQLSAIALAAYSSLAKHGEYG	24	0.603
15	496	510	HGPGPGQQGIVRQRV	15	0.596
16	234	249	FQDGPGPGDRFKRTSF	16	0.585

### Disulfide engineering of the vaccine construct

In the refined model of our vaccine, the DbD2 server predicted 59 residue pairs with the potential to form disulfide bonds. The residue pairs were screened based on χ3 angle (between − 87° and + 97°) and bond energy (less than 2.2 kcal/mol) parameters. Only six residue pairs were chosen for disulfide bond formation after careful consideration, including PHE28-GLY46, THR31-VAL42, PHE32-LYS79, PRO 101-ALA113, PHE284-GLY317, and GLY519-MET549. The original and mutant models of the vaccine candidate are shown in Fig. S5.

### Molecular docking analysis

**Figure 4:**
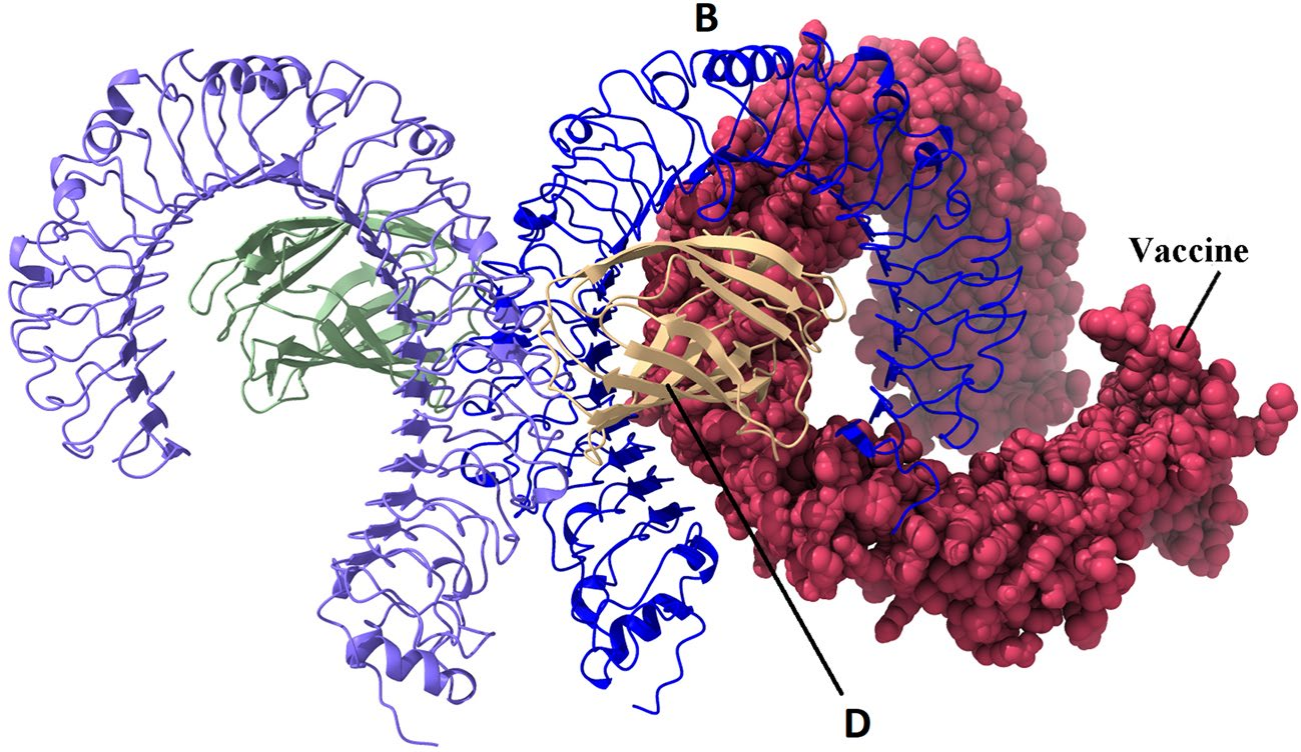
The docked complex of the multi-epitope vaccine and TLR4 was visualized using the UCSF Chimera 1.15rc software. The vaccine construct is shown in the spheres form, while TLR4 is shown in the cartoon form.

**Figure 5:**
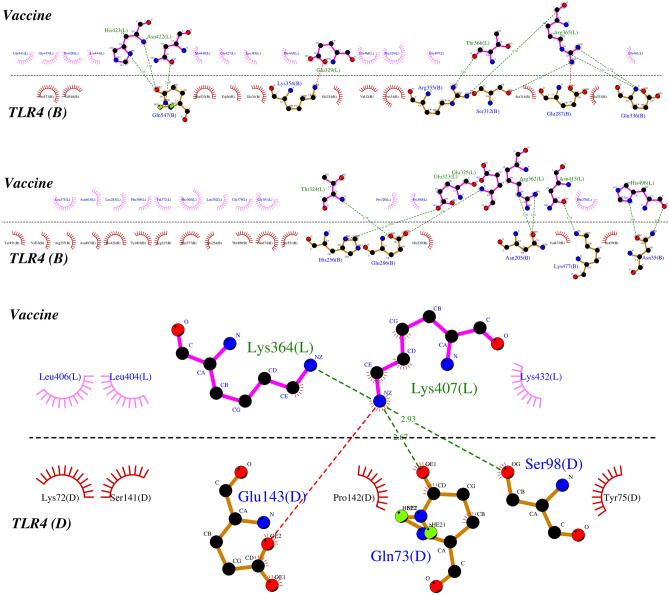
Interaction of the vaccine with chains B and D of TLR4 was visualized using the LigPlot software. The green dashed lines indicate hydrogen bonds.

ClusPro 2.0 server conducted molecular docking of the multi-epitope vaccine and TLR4 and generated 30 docked complexes with different cluster members and the lowest energy. Cluster No. 2 had the lowest energy score of − 1279.1 kcal/mol with 38 members, which was the most negative score (strongest interaction) among all docked complexes (Fig. 4). The residues involved in the interaction between TLR4 and vaccine were analyzed by LigPlot software (Fig. 5). Also, the list of residues with hydrogen bonds along with the bond length is given in Tables 5 and 6.

**Table 5:** List of residues in the docked complex that are involved in hydrogen bonds between the vaccine and TLR4 (chain B).

TLR4 (chain B)	Vaccine	Bond length (Å)
Gln547	His423	2.75
		2.89
	Asn422	2.68
Arg355	Thr366	2.65
	Arg365	2.70
Ser312	Arg365	2.65
Glu336	Arg365	3.01
		2.75
His256	Glu323	3.02
Glu286	Thr324	2.78
	Glu325	2.88
Asn205	Arg362	3.04
		2.87
Lys477	Asn415	2.72
Asn35	His496	2.91
		2.80

**Table 6:** List of residues in the docked complex that are involved in hydrogen bonds between the vaccine and TLR4 (chain D).

TLR4 (chain D)	Vaccine	Bond length (Å)
Gln73	Lys407	2.67
Ser98	Lys364	2.93

### Molecular dynamics simulation of the docked complex

**Figure 6:**
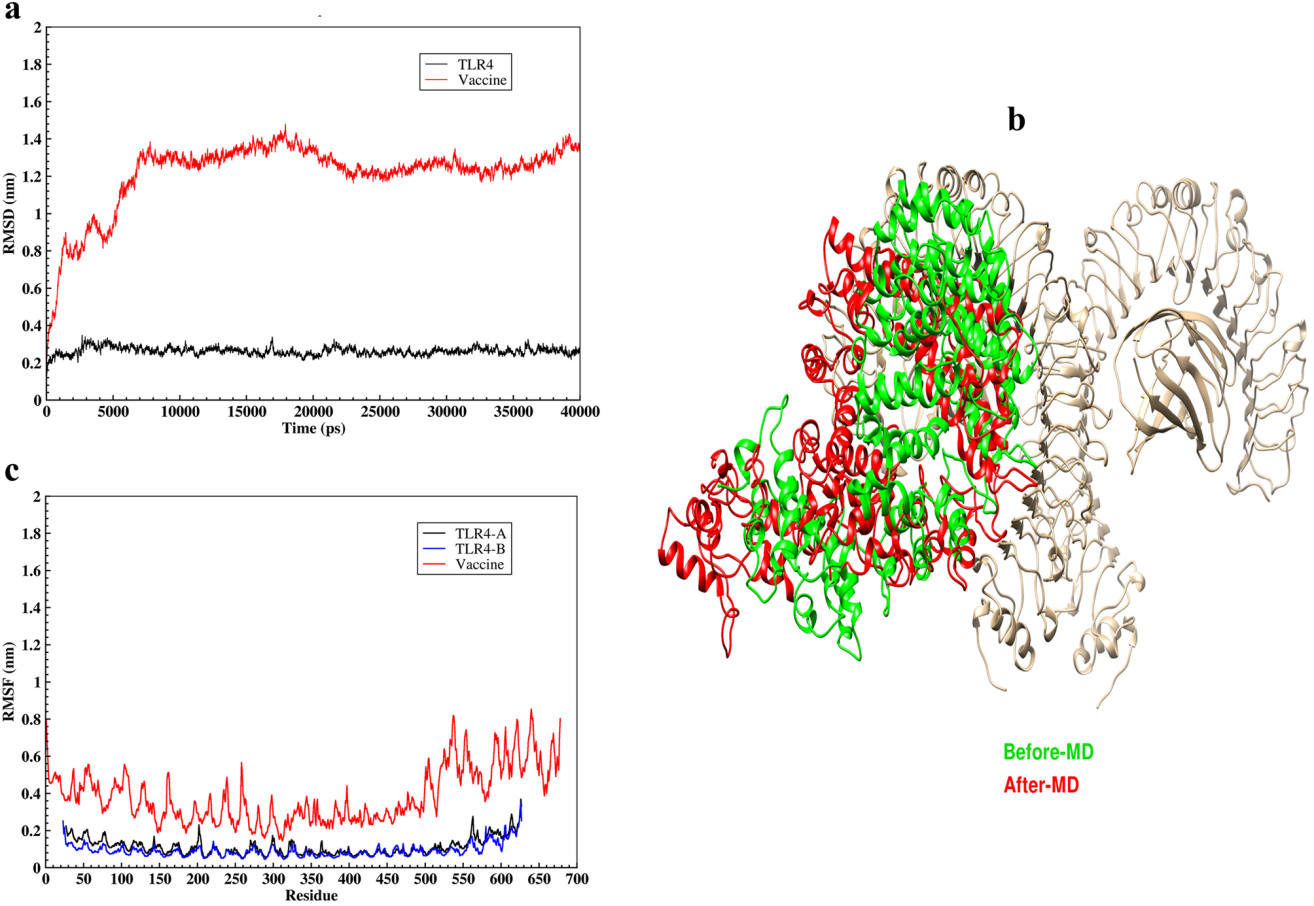
Molecular dynamics simulation analysis of the vaccine–TLR4 complex. (**a**) RMSD plot of the vaccine and TLR 4 in the docked complex for a time duration of 40 ns. (**b**) Conformational changes of the vaccine in the docked complex before (green) and after (red) molecular dynamics simulation was visualized using the UCSF UCSF Chimera 1.15rc software. (**c**) RMSF plot of the vaccine and TLR 4 in the docked complex.

The molecular dynamic simulation of the vaccine-TLR4 complex was performed using GROMACS 2019.6 software for 40 ns. The RMSD graph is used to assess the structure's stability during the simulation. The RMSD value of TLR4 increased rapidly at the beginning of the simulation, reaching about 0.3 nm at 3000 ps and remaining stable until the end of the simulation. An initial sudden change occurred in the RMSD graph of the vaccine and after 6000 ps it has reached the value of 1.3 nm and showed slight fluctuations around this value until the end of the simulation (Fig. 6a). The input and output structures of the molecular dynamics simulation are stacked so that any changes can be detected. After simulating molecular dynamics, the vaccine shows a relatively high conformational change, as shown in Fig. 6b. In molecular dynamics studies, RMSF is the most commonly used method for measuring the oscillating motions of macromolecules. As seen in the previous section, the vaccine is attached to chain B of TLR4, and since the chains A and B have the same sequence on the TLR4, to accurately investigate the effect of vaccine binding on chain B flexibility, the RMSF plot for chains A and B is provided. The flexibility of the residues at both ends of the TLR4 sequence (N-terminal and C-terminal) in chain A was greater than in chain B, while the flexibility of the other regions in the two chains was the same and did not show a notable change. The RMSF plot of the vaccine revealed that the majority of the vaccine's residues had slight flexibility, indicating that the vaccine construct has established stable interactions with TLR4 (Fig. 6c).

### Codon optimization and in silico cloning

**Figure 7:**
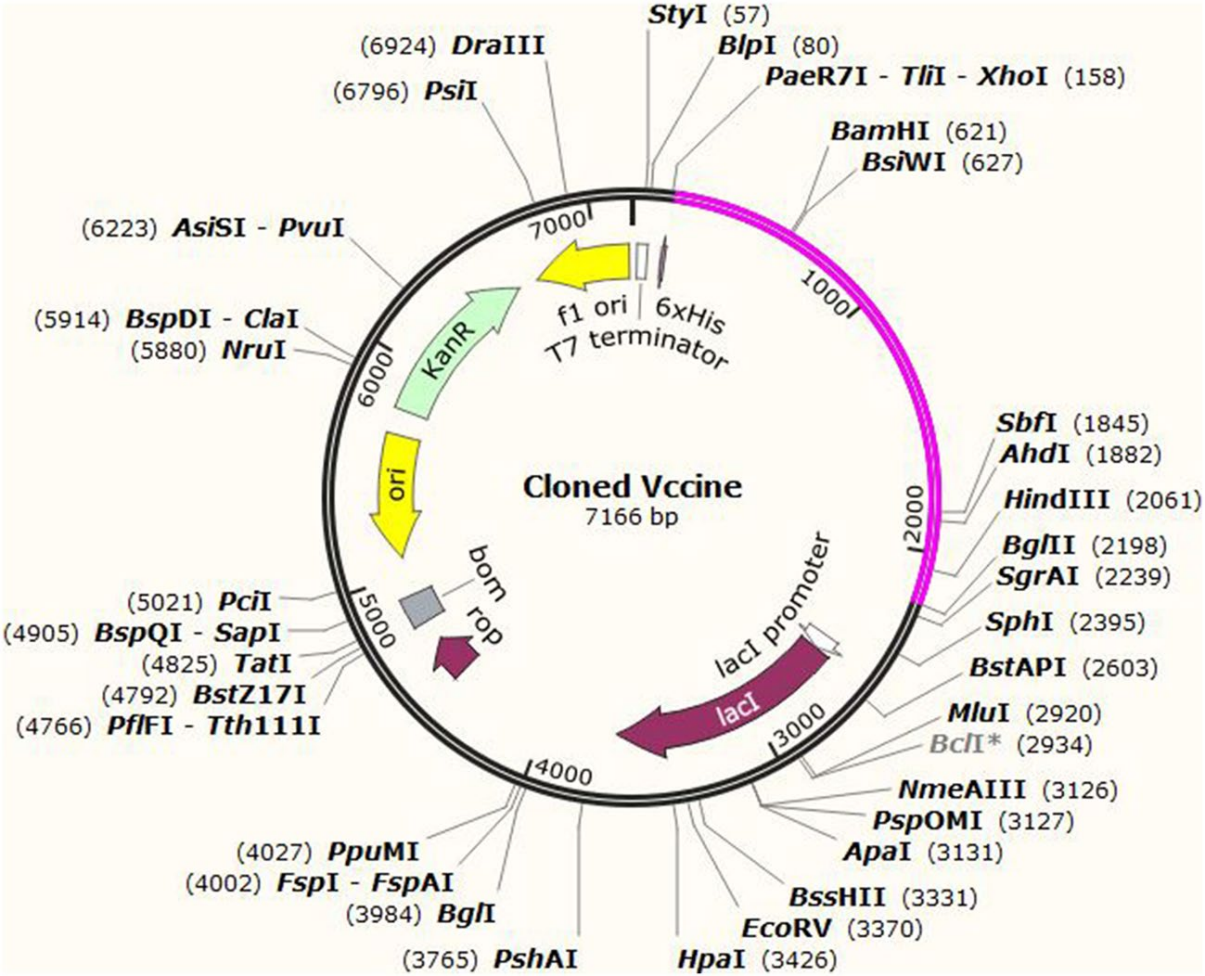
In silico cloning of the vaccine construct between *Xho*I and *Bgl*II restriction sites of pET-28a (+) vector using SnapGene sofware free-trial (https://www.snapgene.com/free-trial/). The magenta part represents the vaccine sequence, while the black part represents the backbone of the pET28a (+) vector.

The vaccine construct was back translated using JCat to a cDNA sequence with a length of 2034 nucleotides. The vaccine sequence had a CAI value of 0.9571 and a GC content of 53.09%. The vaccine sequence was cloned into the pET-28a (+) vector by SnapGene software. The length of the cloned vaccine was estimated to be 7.166 kbp (Fig. 7).

## Discussion

EBOV disease is one of the most lethal viral diseases affecting both humans and non-human mammals. The disease's mortality rate is reported to be 90%^51^. The Ebola outbreak was labeled a public health emergency of international concern by the World Health Organization (WHO) in August 2014^52^. Ebola and Marburg are both members of the *Filoviridae* family, and they are classified as bioterrorism in category A, alongside diseases such as plague, smallpox, and anthrax^5^. Because of their high virulence, demonstrated aerosol infectivity in the laboratory, and ability to induce stress and paranoia, the filoviruses have been classified as high priority Category A pathogens by the Centers for Disease Control and Prevention (CDC, USA)^53^.

Vaccines are a safe and effective way of limiting the spread of lethal infectious diseases and saving millions of lives^54^. Conventional vaccine development methods are labor-intensive, costly, and time-consuming. Furthermore, the likelihood of failure in subsequent trials is high^55^. The reverse vaccinology will employ computational approaches and bioinformatics tools for antigen identification. This method can identify new potential antigenic proteins that play an important role in the immunogenicity and the safety of the vaccines^56^. Recently, reverse vaccinology approaches have been utilized to develop multi-epitope vaccines against dengue virus^57^, human cytomegalovirus^58^, SARS-CoV-2^48,59^, *Mycobacterium tuberculosis*^60^*, Helicobacter pylori*^61^, *Leishmania infantum*^62^, *Echinococcus granulosus*^63^, and *Candida auris*^64^.

In this work, we designed a multi-epitope vaccine against EBOV. The identification of antigenic proteins is an important step in vaccine design. Using computational methods, we conducted an organized and thorough evaluation of the Ebola proteins and selected the VP35, VP24, VP30, VP40, GP, and NP proteins as target proteins for epitope prediction. CTL, HTL, and linear B-cell epitopes were predicted from these proteins. In the first step, epitopes (CTL and HTL epitopes) that interacted with a large number of HLA alleles were chosen for further investigation. To ensure that the selected CTL, HTL, and linear B cell epitopes are sufficiently antigenic and conserved while remaining non-toxic and non-allergenic to vaccine recipients, various immunoinformatics tools were used to screen epitopes for the aforementioned parameters. Aside from the aforementioned parameters, HTL epitopes were tested for their ability to induce IFN-gamma and IL-4 production. IFN-gamma has a crucial function in both adaptive and innate immune activation, in addition to interfering with viral replication^65^. IL-4 causes allergic reactions. This is due to the fact that IL-4 directs the development of TH2, which results in the production of IgE^66^. Different linkers were utilized to connect epitopes with various patterns in several primary structures of vaccine construct. After analyzing the physicochemical characteristics, particularly the stability of the generated constructs, it was found that AAY, GPGPG, and KK linkers are appropriate for joining CTL, HTL, and linear B-cell epitopes, respectively. Our vaccine construct's primary structure included 1 adjuvant, 7 CTL epitopes, 17 HTL epitopes, 6 linear B-cell epitopes, 1 EAAAK linker, 6 AAY linkers, 17 GPGPG linkers, and 6 KK linkers. The EAAAK linker is a rigid linker that, due to its helix formation properties, helped improve the immunogenic properties^43,67^. AAY, GPGPG, and KK linkers are typically made up of flexible and hydrophilic amino acids, which could help to prevent domain disruption^68^.

The vaccine's physicochemical characteristics were evaluated to facilitate subsequent experimental evaluations of the vaccine and to enable the successful setup of in vitro and in vivo assays. The theoretical pI of the vaccine was determined to be 9.09, suggesting that the vaccine is basic in nature. The vaccine construct's GRAVY was − 0.264; a negative value for this parameter indicates that the vaccine is hydrophilic and also has a high degree of solubility^69^. Our vaccine construct has a lower GRAVY than the vaccine designed in the study by Kadam et al.^38^, indicating that our vaccine will interact better with water molecules. The half-life of our vaccine in mammalian, yeast, and *E. coli* was determined to be 30, 20, and 10 h, respectively, implying that the vaccine is exposed to the immune system for a longer period of time and induces more immune responses^70^. The predicted half-life of the vaccine designed in the study of Shankar et al.^40^ was identical to the half-life of our vaccine. The vaccine's molecular weight was determined to be 73.15 kDa; vaccines with molecular weights less than 110 kDa could be a good vaccine candidate since they are easier to clone and express in expression systems than large proteins^71^. The aliphatic index score was 81.47; an aliphatic index greater than 50 indicates that the vaccine is stable at higher temperatures^72^. The vaccine instability index was 26.72, indicating its stability. The instability index of the vaccine designed in the study by Kadam et al.^38^ was 38, indicating that our vaccine is more stable. In general, a protein with an instability index of less than 40 is considered stable^73^. The Solpro server result showed that our vaccine was soluble. The vaccine construct was identified as non-allergen by the AllerTOP v. 2.0 server. Our vaccine had high antigenicity scores on both the ANTIGENpro and the Vaxijen v2.0 servers. The results of predicting the vaccine's second structure were acceptable because the number of helices in the second structure, which indicates the number of hydrogen bonds and thus the protein's stability, was sufficient^74^.

I-TASSER server predicted the vaccine 3D model, which was then refined by the GalaxyRefine server. The Ramachandran plot revealed that 72.29% of the residues in the initial vaccine model were in the favoured region, which increased to 88.76% percent after refining. The initial model had a z-score of − 2.46, whereas the refined model had a z-score of − 3.47. A more negative z-score indicates that the 3D refined model of the vaccine is of higher quality^75^. In the 3D refined model of the vaccine, 16 discontinuous B-cell epitopes were predicted. Because discontinuous B-cell epitopes play a critical role in humoral immune responses by producing antibodies^76^, our designed vaccine has the potential to induce large amounts of antibody production. The vaccine's refined structure was subjected to disulfide engineering, and 6 disulfide bonds were introduced in the refined model to increase the vaccine structure's stability^77^.

Since TLR4 is involved in the activation of proinflammatory mediators after EBOV infection^78^, the molecular docking analysis of the vaccine construct with TLR4 was carried out. The results of this analysis revealed that the vaccine had a significant affinity for TLR4, implying that it may elicit both an innate and adaptive immune response^79^. The MD simulation results of the vaccine-TLR4 docked complex revealed that the vaccine reached a stable state in less than 40 ns and that its flexible residues were present in the range of 500–678, because this region had no interaction with TLR4, and can move freely, so its flexibility is higher than other regions of the vaccine construct. JCat server revealed that the vaccine construct had a CAI of 0.9571 and a GC content of 53.09%. CAI greater than 0.890^80^ and GC content between 30 and 70%^76^ is ideal for target organism expression. In the present study, we used a set of bioinformatics software to design a multi-epitope vaccine against Ebola, and the results were promising. The only limitation of this study is the need for further in vitro and in vivo studies to demonstrate the vaccine candidate's efficacy and safety.

## Conclusion

EBOV is one of the most dangerous viruses among viral hemorrhagic fevers. The purpose of the current study was to design a multi-epitope vaccine against EBOV utilizing immunoinformatics approaches. T-cell and B-cell epitopes of target antigens were predicted, and epitope screening was conducted correctly and sequentially. The vaccine possessed all of the desirable qualities of a vaccine candidate, including good physicochemical properties, solubility, high antigenicity, and non-allergenicity. The vaccine's strong affinity for TLR4 was validated by molecular docking analyses, and the vaccine's stability was ensured by MD simulation. The codon optimization also presented an optimistic CAI value and GC content, confirming the vaccine's expression in a bacterial host. The rational design of the linear structure of the multi-epitope vaccine, which resulted from the proper arrangement of the selected epitopes and adjuvant, made the results of this study more impressive than previous studies. Our findings suggest that the vaccine candidate may elicit appropriate immune responses. As a result, we believe that this vaccine candidate, if further evaluated in vitro and in vivo, could be a promising vaccine against EBOV.

## Materials and methods

### Identification and retrieval of viral protein sequences

In the first step of the study, antigen proteins of the Zaire Ebola virus were identified using literature^38,39,81^ and the Protegen server (http://www.violinet.org/protegen/). The Protegen is a web-based central database and processing system for collecting, storing, and analyzing protective antigens^82^. The reference sequences of the identified proteins were retrieved in FASTA format from the NCBI database (https://www.ncbi.nlm.nih.gov/), and their antigenicity was evaluated using the VaxiJen v2.0 server (http://www.ddg-pharmfac.net/vaxijen/VaxiJen/VaxiJen.html). VaxiJen is the first server for alignment-independent prediction of antigenic peptides or proteins from a variety of organisms such as bacteria, viruses, parasites, fungi, and tumors^83–85^.

### T-cell epitope prediction

CTL epitopes were predicted for the selected proteins using the NetCTL 1.2 server (http://www.cbs.dtu.dk/services/NetCTL/). The server predicts CTL epitopes (9-mer) for 12 MHC class I supertypes, including A1, A2, A3, A24, A26, B7, B8, B27, B39, B44, B58, and B62. This server identifies epitopes based on proteasomal C terminal cleavage, MHC class I binding peptide, and TAP transport efficiency scores^86^. For the present study 12 MHC class I supertypes were selected to predict CTL epitopes, and the cutoff value for epitopes prediction was set at 0.75.

The NetMHCII 2.3 server (http://www.cbs.dtu.dk/services/NetMHCII/) was used to predict HTL epitopes. The NetMHCII 2.3 server identifies the binding of the peptides to the HLA-DR, HLA-DQ, HLA-DP, and mouse MHC class II alleles using artificial neural networks (ANNs)^87^. The threshold values corresponding to the strong and weak binder were set at 2% and 10%, respectively.

The predicted CTL and HTL epitopes were analyzed for antigenicity, toxicity, allergenicity, and conservancy. The antigenicity of the epitopes was checked with the help of the VaxiJen v2.0 server at a threshold value of 0.4. The toxicity or non-toxicity of the epitopes was determined by the ToxinPred server (https://webs.iiitd.edu.in/raghava/toxinpred/design.php) using the SVM (Swiss-Prot) based method^88^. The AllerTOP v. 2.0 server (https://www.ddg-pharmfac.net/AllerTOP/method.html) was used to analyze the allergenicity of the epitopes. AllerTOP is an alignment-free server for allergenicity prediction based on protein physicochemical parameters^89^. The Epitope Conservancy Analysis tool from the Immune Epitope Database (IEDB) (http://tools.iedb.org/conservancy/) was used to determine the degree of the conservancy of predicted epitopes. This tool calculates the degree of conservation of an epitope within a defined protein sequence^90^. In addition to the above mentioned screenings, HTL epitopes were checked for IFN-gamma and IL-4 production. The IFNepitope server (http://crdd.osdd.net/raghava/ifnepitope/design.php)^91^ and the IL4pred server (http://crdd.osdd.net/raghava/il4pred/)^92^ were used to predict the ability of HTL epitopes to induce IFN-gamma and IL-4, respectively.

### Linear B-cell epitope prediction

The Antigen Sequence Properties tool from IEDB (http://tools.iedb.org/bcell/) was used to predict linear B-cell epitopes. The epitopes were predicted using the Bepipred Linear Epitope Prediction 2.0 method^93^. Finally, these predicted epitopes were assessed for antigenicity, toxicity, allergenicity, and conservancy using VaxiJen v2.0, ToxinPred, AllerTOP v. 2.0, and Epitope Conservancy Analysis, respectively.

### Multi-epitope vaccine construction

To construct a multi-epitope vaccine, the selected CTL, HTL, and linear B-cell epitopes were incorporated using suitable linkers. AAY (Ala-Ala-Tyr), GPGPG (Gly-Pro-Gly-Pro-Gly), and KK (bi-lysine) linkers were used to connect the CTL, HTL, and linear B-cell epitopes, respectively. These linkers were added to achieve the successful separation of individual epitopes^94^. Finally, to improve the immunogenicity of the multi-epitope vaccine, 50S ribosomal L7/L12 (Locus RL7_MYCTU) sequence with accession no. P9WHE3 as an adjuvant was added to the N-terminal of the vaccine construct via an EAAAK linker.

### Evaluating physicochemical, solubility, allergenicity, and antigenicity parameters of the designed vaccine

The ProtParam tool of the Expasy server (https://web.expasy.org/protparam/) was used to analyze a set of physicochemical parameters of the multi-epitope vaccine, including theoretical pI, grand average of hydropathicity (GRAVY), half-life, molecular weight, aliphatic index, and instability index^95^. The solubility of the proposed vaccine was predicted by the SOLpro server (http://scratch.proteomics.ics.uci.edu/). The SOLpro uses a two-stage SVM algorithm based on multiple representations of the primary sequence to predict whether a protein would be soluble when overexpressed in *Escherichia coli* (*E. coli*)^96^. AllerTOP v. 2.0 server was utilized to evaluate the allergenicity of the multi-epitope vaccine. The antigenicity of the proposed vaccine was checked by ANTIGENpro and VaxiJen v2.0 servers. ANTIGENpro server (http://scratch.proteomics.ics.uci.edu/) predicts the antigenicity of proteins or peptides using a two-stage architecture of their sequence and five machine learning algorithms^97^.

### Secondary structure prediction

PDBsum (http://www.ebi.ac.uk/thornton-srv/databases/cgi-bin/pdbsum/GetPage.pl?pdbcode=index.html) was employed to predict the secondary structure of the proposed vaccine. PDBsum is a web server that provides information on the protein secondary structure, protein–ligand, protein-DNA interactions, and protein structure quality^98^.

### Tertiary structure prediction, refinement, and validation of the vaccine construct

The tertiary structure of the multi-epitope vaccine construct was modeled using the I-TASSER server (https://zhanggroup.org/I-TASSER/). I-TASSER server builds 3D structures from the amino acid sequence by reconfiguring the excised sections from the threading templates and computes the C-score to determine the correctness of the predicted models^47,99,100^. The GalaxyRefne server (http://galaxy.seoklab.org/cgi-bin/submit.cgi?type=REFINE) then refined the selected 3D model of the vaccine. The GalaxyRefine server employs a refining approach that was validated in CASP10. After reconstructing and repacking the side chains, this approach uses molecular dynamics simulation to achieve a general relaxation of the three-dimensional structure. Based on the CASP10 evaluation, this method performed the best in terms of improving the quality of the local structure^101,102^. The SWISS-MODEL Structure Assessment (https://swissmodel.expasy.org/assess) and ProSA-web server (https://prosa.services.came.sbg.ac.at/prosa.php) were used to compare the quality of the initial 3D structure and the refined structure of the vaccine. The Ramachandran plot is obtained using SWISS-MODEL Structure Assessment^103^. The Ramachandran plot illustrates the allowed and disallowed dihedral angles psi (ψ) and phi (ϕ) of amino acid, which are calculated based on the van der Waals radius of the side chain^104^. ProSA web server estimates the overall quality of the model as a z-score. If this z-score falls outside of the normal range for native proteins, the structure most likely contains errors^105,106^.

### Discontinuous B-cell epitope prediction

The ElliPro server (http://tools.iedb.org/ellipro/) was utilized to identify discontinuous B-cell epitopes in the refined 3D model of the vaccine. ElliPro assigns a score to each predicted epitope, which is characterized as a Protrusion Index (PI) value averaged over epitope residues^107^. The minimum score in this analysis was set at 0.5, while the maximum distance was set at 6 Å.

### Disulfide engineering of the vaccine construct

Disulfide engineering is a new strategy of designing new disulfide bonds in the target protein through cysteine mutation of protein structure residues, resulting in increased protein structure stability^108^. Therefore, the Disulfide by Design 2 (DbD2) server (http://cptweb.cpt.wayne.edu/DbD2/) was used to identify residue pairs that had the potential for mutation and could be used in disulfide engineering^109^.

### Molecular docking analysis

Molecular docking is a computational approach that predicts the preferred orientation of the ligand to the receptor when they are joined together to form a stable complex^110^. We used the ClusPro 2.0 server (https://cluspro.org/login.php) to perform molecular docking^111–114^. Initially, the 3D structure of TLR4 in PDB format (PDB ID: 4G8A) was obtained from the protein data bank (RCSB). The ligands attached to the retrieved TLR4 structure were then removed using Chimera 1.15rc software. The refined 3D model of the multi-epitope vaccine and TLR4 was submitted in the ClusPro 2.0 server as ligand and receptor, respectively. The LigPlot software was also used to analyze the bonds established between the ligand and receptor residues in the docked complex^115^.

### Molecular dynamics simulation of the docked complex

MD simulation is an effective method for studying ligand and receptor stability in a docked complex at the microscopic level^116^. In this study, MD simulation was run for 40 ns by GROMACS 2019.6 software^117^. Firstly, the input structure was prepared using the ff99SB force field. Following that, Na^+^ and Cl^−^ ions were introduced into the system to neutralize the net charge of the system. The complex was then solvated into a 10 Å layer of TIP3P using the gmx solvate software. The energy minimization of the solvated system was performed using the steepest descent approach to eliminate steric clashes. The system was gradually heated to 300 K for 20 ps and then the system was equilibrated at constant pressure, and the SHAKE algorithm was used to keep the hydrogen bond restrained. Finally, the root mean square deviation (RMSD) and root mean square fluctuation (RMSF) were generated using the gmx rms and gmx rmsf modules, respectively.

### Codon optimization and in silico cloning

The Java Codon Adaptation Tool (JCat) (http://www.jcat.de/) was utilized for reverse translation, codon optimization, and calculating the codon adaptation index (CAI) value and GC content of the vaccine construct in *E. coli* (strain K12)^118^. Three options were chosen here: avoid rho-independent transcription terminators, avoid prokaryotic ribosome binding sites, and avoid cleavage sites of restriction enzyme. The *Xho*I and *Bgl*II restriction sites were tagged at the 5′ and 3′ ends of the vaccine's DNA sequence, respectively. Finally, using SnapGene software (https://www.snapgene.com/free-trial/), the obtained sequence was inserted into the pET28a (+) expression vector between the *Xho*I and *Bgl*II restriction sites.

## Supplementary Information

Supplementary Figures.

Supplementary Tables.
