# Supplementary material for: Designing a novel multi‑epitope vaccine against Ebola virus using reverse vaccinology approach
Source: Sci Rep. 2022 May 11;12:7757. doi: 10.1038/s41598-022-11851-z (PMC9094136; doi:10.1038/s41598-022-11851-z)
Supplement: Supplementary file 1 — Supplementary Figures. [file 41598_2022_11851_MOESM1_ESM.docx]

**Designing a novel multi‑epitope vaccine against Ebola virus using reverse vaccinology approach**

Morteza Alizadeh^a^, Hossein Amini-Khoei^b^, Shahram Tahmasebian^c^, Mahdi Ghatreh-Samani^d^, Keihan Ghatreh-Samani^e^, Yadolah Edalatpanah ^f^, Susan Rostampur ^g^, Majid Salehi^a^, Maryam Ghasemi-Dehnoo^b^, Fatemeh Azadegan-Dehkordi^b^, Samira Sanami^b*^, Nader Bagheri^b^*

^a^ Department of Tissue Engineering, School of Medicine, Shahroud University of Medical Sciences, Shahroud, Iran

^b^ Medical Plants Research Center, Basic Health Sciences Institute, Shahrekord University of Medical Sciences, Shahrekord, Iran

^c^ Department of Medical Biotechnology, School of Advanced Technologies, Shahrekord University of Medical Sciences, Shahrekord, Iran

^d^ Department of Microbiology and Immunology, Cellular and Molecular Research center, Faculty of Medicine, Shahrekord University of Medical Sciences, Shahrekord, Iran

^e^ Clinical Biochemistry Research Center, Shahrekord University of Medical Sciences, Shahrekord, Iran

^f^ Cellular and Molecular Research Center, Yasuj University of Medical Sciences, Iran

^g^ Department of Molecular Medicine, School of Advanced Medical Science and Technology, Shiraz University of Medical Science, Shiraz, Iran

***Corresponding authors**:

Medical Plants Research Center, Basic Health Sciences Institute, Shahrekord University of Medical Sciences, Shahrekord, Iran. Tel: +989302130048; Fax: +98-3813330709. E-mail addresses: samirasanami34@yahoo.com

Medical Plants Research Center, Basic Health Sciences Institute, Shahrekord University of Medical Sciences, Shahrekord, Iran. Tel: +98 9181731073; Fax: +98-3813330709. E-mail addresses: n.bagheri1985@gmail.com

**Supplementary figures**


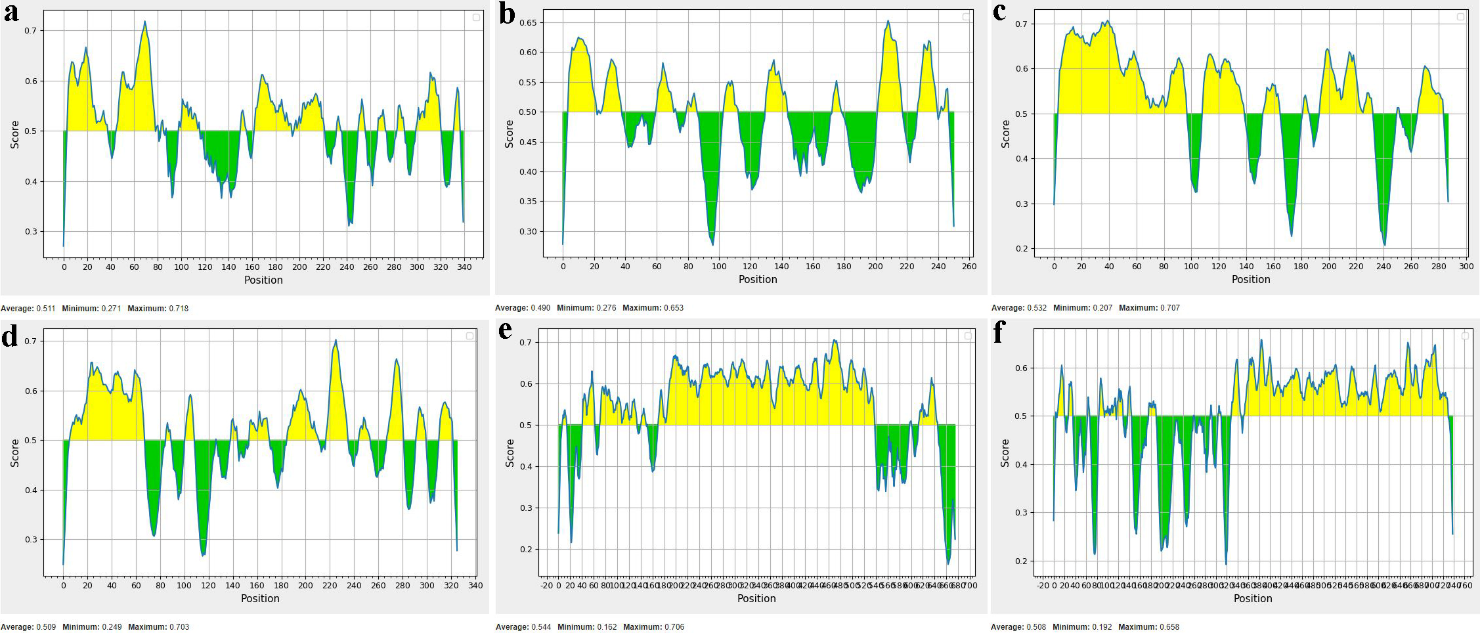


**Figure S1.** The predicted linear B-cell epitopes from (a) VP35, (b) VP24, (c) VP30, (d) VP40, (e) GP, and (f) NP. The yellow regions represent epitopes, while the green regions represent non-epitopes.


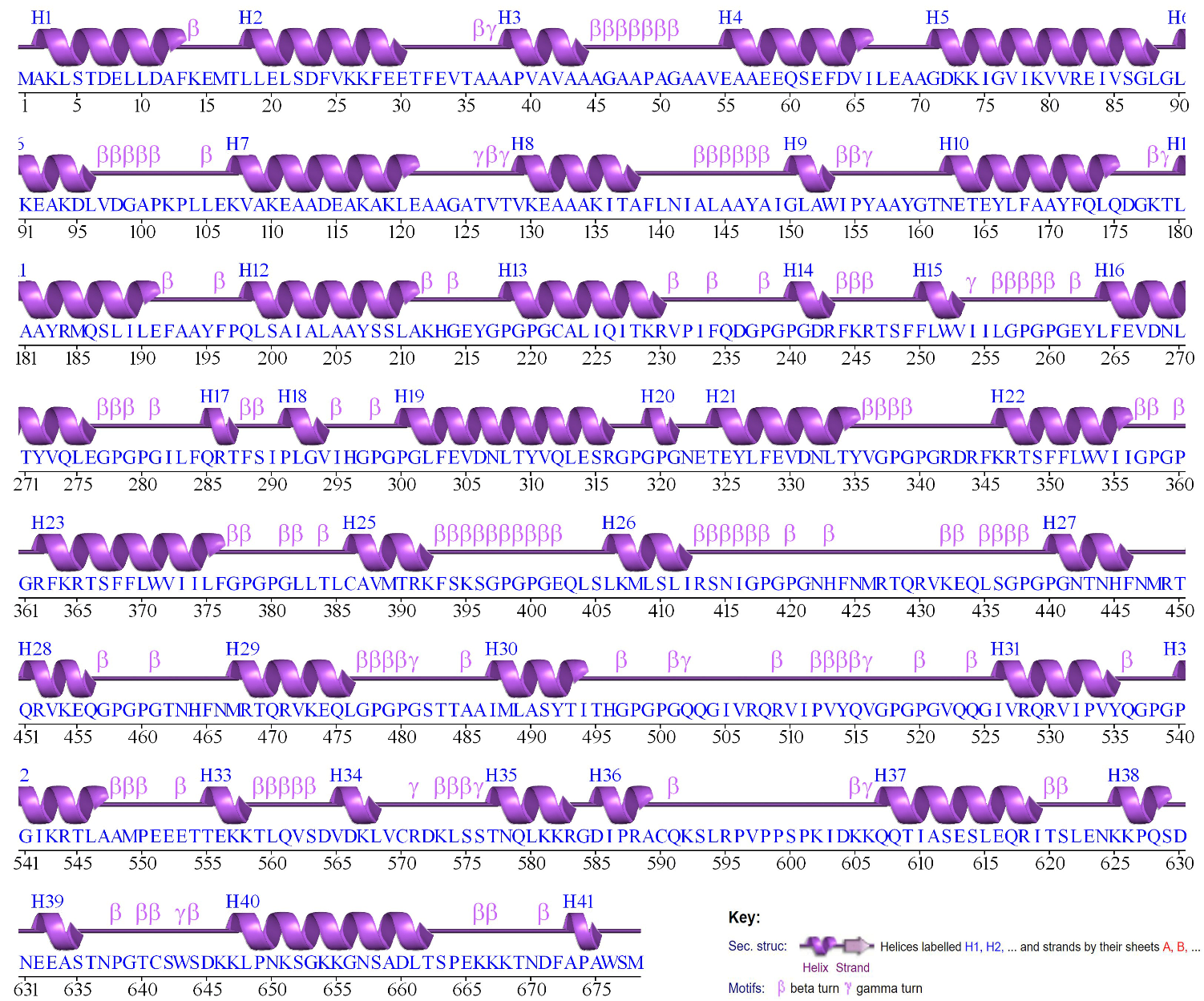


**Figure S2.** The predicted secondary structure of the multi-epitope vaccine.


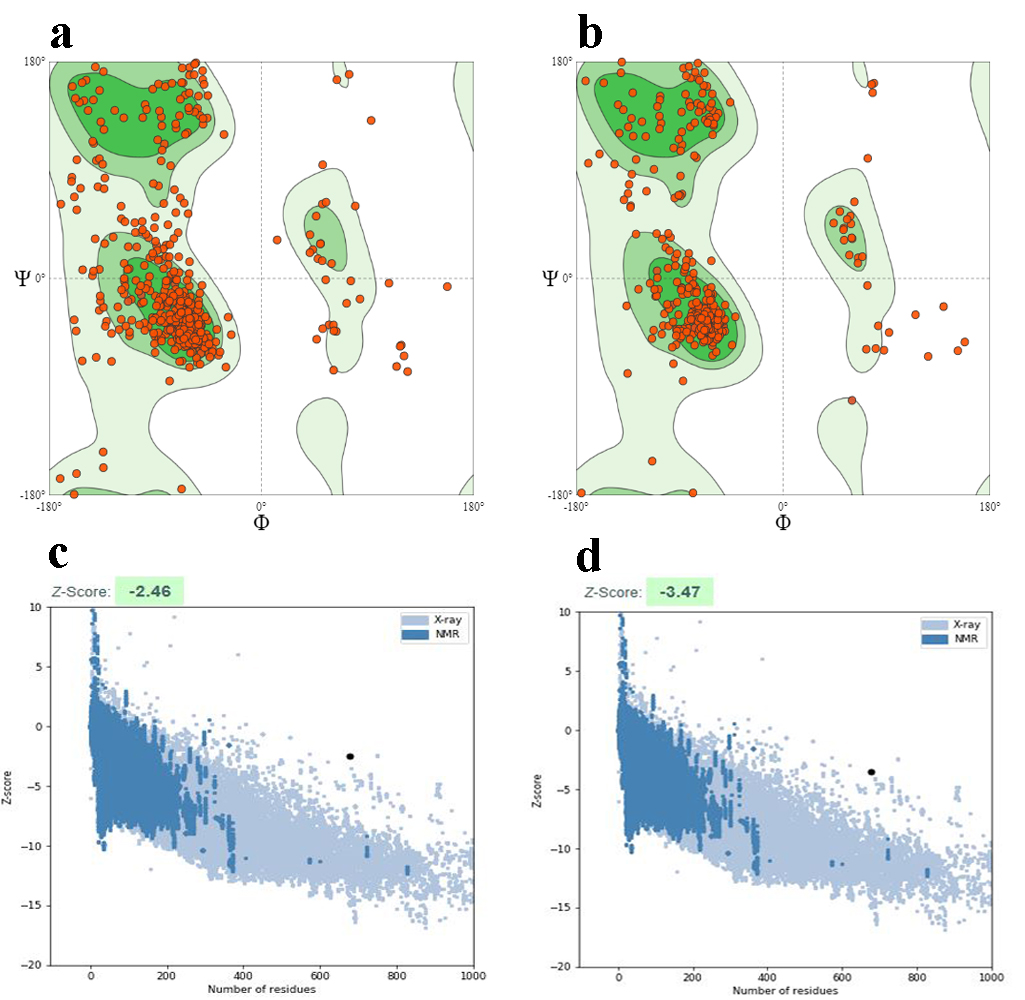


**Figure S3.** Validation of the vaccine construct's 3D model before and after refinement using the Ramachandran plot and ProSA-web. Ramachandran plot shows that the proportion of residues in the favoured region of the (a) initial model and (b) the refined model is 72.29% and 88.76%, respectively. (c) The Z-score of the initial model is -2.46 (d) while the z-score of the refined model is -3.47.


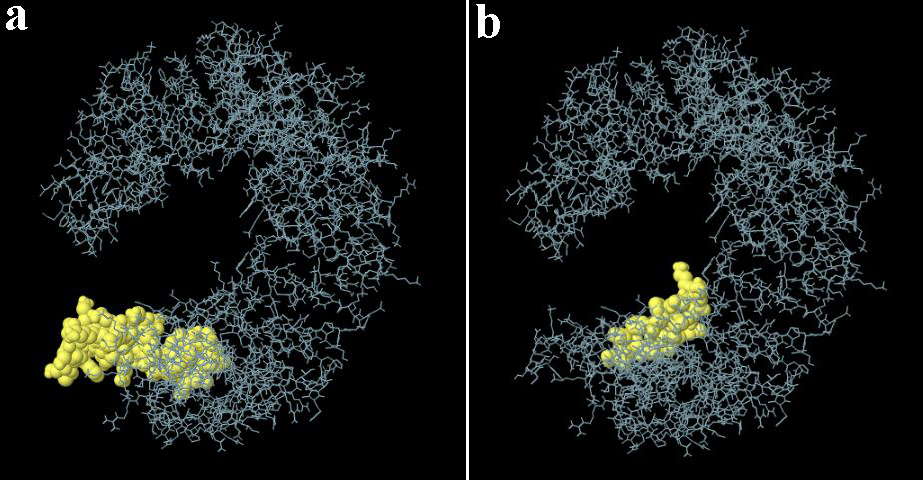


**Figure S4.** The three-dimensional model of the discontinuous B-cell epitopes with a score above 0.8 in the refined model of the vaccine construct. The yellow surfaces are the discontinuous B-cell epitopes and the gray sticks are the rest of the residues. (a) 39 residues with a score of 0.829. (b) 16 residues with a score of 0.812.


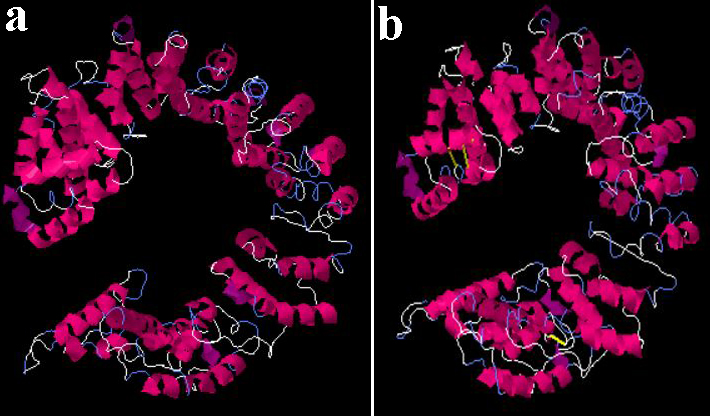


**Figure S5.** Disulfide engineering of the 3D model of the vaccine. (a) Original model (b) mutant model.
