# Supplementary material for: Designing a novel multi‑epitope vaccine against Ebola virus using reverse vaccinology approach
Source: Sci Rep. 2022 May 11;12:7757. doi: 10.1038/s41598-022-11851-z (PMC9094136; doi:10.1038/s41598-022-11851-z)
Supplement: Supplementary file 2 — Supplementary Tables. [file 41598_2022_11851_MOESM2_ESM.docx]

**Designing a novel multi‑epitope vaccine against Ebola virus using reverse vaccinology approach**

Morteza Alizadeh^a^, Hossein Amini-Khoei^b^, Shahram Tahmasebian^c^, Mahdi Ghatreh-Samani^d^, Keihan Ghatreh-Samani^e^, Yadolah Edalatpanah ^f^, Susan Rostampur ^g^, Majid Salehi^a^, Maryam Ghasemi-Dehnoo^b^, Fatemeh Azadegan-Dehkordi^b^, Samira Sanami^b*^, Nader Bagheri^b^*

^a^ Department of Tissue Engineering, School of Medicine, Shahroud University of Medical Sciences, Shahroud, Iran

^b^ Medical Plants Research Center, Basic Health Sciences Institute, Shahrekord University of Medical Sciences, Shahrekord, Iran

^c^ Department of Medical Biotechnology, School of Advanced Technologies, Shahrekord University of Medical Sciences, Shahrekord, Iran

^d^ Department of Microbiology and Immunology, Cellular and Molecular Research center, Faculty of Medicine, Shahrekord University of Medical Sciences, Shahrekord, Iran

^e^ Clinical Biochemistry Research Center, Shahrekord University of Medical Sciences, Shahrekord, Iran

^f^ Cellular and Molecular Research Center, Yasuj University of Medical Sciences, Iran

^g^ Department of Molecular Medicine, School of Advanced Medical Science and Technology, Shiraz University of Medical Science, Shiraz, Iran

***Corresponding authors**:

Medical Plants Research Center, Basic Health Sciences Institute, Shahrekord University of Medical Sciences, Shahrekord, Iran. Tel: +989302130048; Fax: +98-3813330709. E-mail addresses: samirasanami34@yahoo.com

Medical Plants Research Center, Basic Health Sciences Institute, Shahrekord University of Medical Sciences, Shahrekord, Iran. Tel: +98 9181731073; Fax: +98-3813330709. E-mail addresses: n.bagheri1985@gmail.com

**Supplementary tables**

Table S1

Results of CTL epitope prediction of VP35 protein from EBOV.

| **Epitope** | **Position** | **Allels** | **Antigenicity** | **Toxicity** | **Allergenicity** | **Conservancy** |
| --- | --- | --- | --- | --- | --- | --- |
| ATAAATEAY | 154-162 | A1, A26, B58, B62 | 0.5105 | Non-toxin | Allergen | 98.89% |
| IMYDHLPGF | 227-235 | A2, A26, B58, B62 | 0.0731 | Non-toxin | Non-allergen | 98.89% |
| **FQLQDGKTL** | **328-336** | **B39, B44, B62** | **0.7405** | **Non-toxin** | **Non-allergen** | **93.33%** |
| HLPGFGTAF | 231-239 | A26, B8, B62 | -0.6065 | Non-toxin | Non-allergen | 98.89% |

The selected epitopes have been shown in bold.

Table S2

Results of CTL epitope prediction of VP24 protein from EBOV.

| **Epitope** | **Position** | **Allels** | **Antigenicity** | **Toxicity** | **Allergenicity** | **Conservancy** |
| --- | --- | --- | --- | --- | --- | --- |
| KVYWAGIEF | 39-47 | B7, B58, B62 | 1.2265 | Non-toxin | Allergen | 92.16% |
| QTIQGWKVY | 33-41 | A1, A26, B58, B62 | -0.2729 | Non-toxin | Allergen | 78.43% |
| RMKPGPAKF | 211-219 | B8, B58, B62 | 0.7439 | Non-toxin | Allergen | 11.76% |
| **RMQSLILEF** | **237-245** | **A1, A24, B8, B27, B58, B62** | **0.7883** | **Non-toxin** | **Non-allergen** | **92.16%** |
| TTNTNHFNM | 128-136 | A1, A26, B58 | 0.8669 | Non-toxin | Allergen | 96.08% |

The selected epitopes have been shown in bold.

Table S3

| **Epitope** | **Position** | **Allels** | **Antigenicity** | **Toxicity** | **Allergenicity** | **Conservancy** |
| --- | --- | --- | --- | --- | --- | --- |
| **ITAFLNIAL** | **239-247** | **B8, B39, B62** | **0.5463** | **Non-toxin** | **Non-allergen** | **98.08%** |
| QAEPVLEVY | 203-211 | A1, B58, B62 | 0.5986 | Non-toxin | Allergen | 100.00% |
| SQVRVPTVF | 46-54 | A24, A27, B39, B58, B62 | -0.3704 | Non-toxin | Non-allergen | 96.15% |

Results of CTL epitope prediction of VP30 protein from EBOV.

The selected epitopes have been shown in bold

Table S4

| **Epitope** | **Position** | **Allels** | **Antigenicity** | **Toxicity** | **Allergenicity** | **Conservancy** |
| --- | --- | --- | --- | --- | --- | --- |
| KIQAIMTSL | 236-244 | A2, B7, B8 | 0.5797 | Non-toxin | Allergen | 98.68% |
| KQIPIWLPL | 90-98 | A2, A27, B39, B44, B58, B62 | 0.1752 | Non-toxin | Non-allergen | 98.68% |
| **TAAIMLASY** | **112-120** | **A1, A26, B58, B62** | **0.4648** | **Non-toxin** | **Non-allergen** | **97.37%** |
| VQLPQYFTF | 166-174 | A24,B27, B58, B62 | 0.3562 | Non-toxin | Non-allergen | 94.74% |

Results of CTL epitope prediction of VP40 protein from EBOV.

The selected epitopes have been shown in bold.

Table S5

Results of CTL epitope prediction of GP protein from EBOV.

| **Epitope** | **Position** | **Allels** | **Antigenicity** | **Toxicity** | **Allergenicity** | **Conservancy** |
| --- | --- | --- | --- | --- | --- | --- |
| **AIGLAWIPY** | **526-534** | **A1, A3, A26, B62** | **1.5763** | **Non-toxin** | **Non-allergen** | **100.00%** |
| ATEDPSSGY | 205-213 | A1, A26, B62 | 0.27 | Non-toxin | Allergen | 95.24% |
| **FFLWVIILF** | **19-27** | **A24, B8, B62** | **0.4561** | **Non-toxin** | **Non-allergen** | **100.00%** |
| FQRTFSIPL | 27-35 | A2, B8, B27, B39, B44, B62 | 0.2763 | Non-toxin | Non-allergen | 98.81% |
| **GTNETEYLF** | **226-234** | **A1, B58, B62** | **0.7059** | **Non-toxin** | **Non-allergen** | **100.00%** |
| LANETTQAL | 561-569 | B7, B39, B62 | 0.4009 | Non-toxin | Allergen | 100.00% |
| MASENSSAM | 342-350 | B7, B8, B62 | 0.3378 | Non-toxin | Non-allergen | 98.81% |
| NSTHNTPVY | 386-394 | A1, A26, B62 | 0.1792 | Non-toxin | Allergen | 96.43% |
| **RTSFFLWVI** | **16-24** | **A1, A2, A24, B58** | **0.7129** | **Non-toxin** | **Non-allergen** | **97.62%** |
| TFAEGVVAF | 175-183 | A24, B39, B62 | 0.0888 | Non-toxin | Non-allergen | 98.81% |
| TVIYRGTTF | 168-176 | A24, A26, B7, B8, B58, B62 | 0.4593 | Non-toxin | Allergen | 100.00% |
| VIIAVIALF | 661-669 | A24, A26, B58, B62 | 0.2476 | Non-toxin | Non-allergen | 100.00% |

The selected epitopes have been shown in bold.

Table S6

Results of CTL epitope prediction of NP protein from EBOV.

| **Epitope** | **Position** | **Allels** | **Antigenicity** | **Toxicity** | **Allergenicity** | **Conservancy** |
| --- | --- | --- | --- | --- | --- | --- |
| FEEMYRHIL | 648-656 | B8, B39, B44 | -0.6302 | Non-toxin | Non-allergen | 96.77% |
| **FPQLSAIAL** | **313-321** | **B7, B8, B39** | **1.101** | **Non-toxin** | **Non-allergen** | **100.00%** |
| FRLMRTNFL | 201-209 | B8, B27, B39 | 0.2432 | Non-toxin | Non-allergen | 100.00% |
| GQFLSFASL | 148-156 | A2, B27, B39, B44, B62 | 0.7713 | Non-toxin | Allergen | 100.00% |
| GSYGEYQSY | 464-472 | A1, A3, B58, B62 | 1.0737 | Non-toxin | Allergen | 55.91% |
| LMLCLHHAY | 75-83 | A1, A3, B8, B62 | 0.7446 | Non-toxin | Allergen | 100.00% |
| QQTNAMVTL | 389-397 | B27, B39, B44, B62 | 0.6004 | Non-toxin | Allergen | 100.00% |
| RLAKLTEAI | 401-409 | A2, B7, B8 | 0.025 | Non-toxin | Allergen | 96.77% |
| RLMRTNFLI | 202-210 | A2, A3, A24, B58 | 0.2174 | Non-toxin | Allergen | 100.00% |
| SADSFLLML | 69-77 | A1, A2, B39, B44 | -0.0931 | Non-toxin | Allergen | 97.85% |
| SLPKTSGHY | 413-421 | A1, A3, A26, B62 | 0.0523 | Non-toxin | Allergen | 91.40% |
| SLTESDMDY | 13-21 | A1, A26, B62 | 1.3007 | Non-toxin | Allergen | 70.97% |
| **SSLAKHGEY** | **285-293** | **A1, B58, B62** | **0.4575** | **Non-toxin** | **Non-allergen** | **97.85%** |
| TVAPPAPVY | 603-611 | A1, A3, A26, B58, B62 | 0.2633 | Non-toxin | Allergen | 96.77% |
| VTLDGQQFY | 713-721 | A1, A3, A26, B62 | -0.0349 | Non-toxin | Non-allergen | 98.92% |
| YLEGHGFRF | 98-106 | A1, A26, B62 | -0.9846 | Non-toxin | Allergen | 98.92% |
| YQGDYKLFL | 83-91 | A2, B39, B44, B62 | -0.374 | Non-toxin | Allergen | 87.10% |

The selected epitopes have been shown in bold.

Table S7

Results of HTL epitope prediction of VP35 protein from EBOV

| **Epitope** | **Position** | **Alleles** | **Antigenicity** | **Toxicity** | **Allergenicity** | **Conservancy** | **IFN-gamma** | **IL-4** |
| --- | --- | --- | --- | --- | --- | --- | --- | --- |
| MTTGRATATAAATEA | 147-161 | HLA-DQA10201-DQB10301, HLA-DQA10201-DQB10303, HLA-DQA10501-DQB10301, HLA-DQA10501-DQB10302, HLA-DQA10501-DQB10303 | 0.7755 | Non-toxin | Allergen | 96.67% | Positive | Inducer |
| TTGRATATAAATEAY | 148-162 | HLA-DQA10201-DQB10301, HLA-DQA10201-DQB10303, HLA-DQA10301-DQB10301, HLA-DQA10501-DQB10301, HLA-DQA10501-DQB10302, HLA-DQA10501-DQB10303 | 0.687 | Non-toxin | Allergen | 97.78% | Positive | Inducer |
| GRATATAAATEAYWA | 150-164 | HLA-DQA10102-DQB10602, HLA-DQA10201-DQB10301, HLA-DQA10201-DQB10303, HLA-DQA10301-DQB10301, HLA-DQA10501-DQB10301, HLA-DQA10501-DQB10302, HLA-DQA10501-DQB10303 | 0.8477 | Non-toxin | Allergen | 96.67% | Positive | Inducer |
| QCALIQITKRVPIFQ | 274-288 | DRB1_0103, DRB1_0801, DRB1_0802, DRB1_1101, DRB1_1301, DRB4_0103 | 0.4847 | Non-toxin | Allergen | 94.44% | Positive | Inducer |
| TGRATATAAATEAYW | 149-163 | HLA-DQA10201-DQB10301, HLA-DQA10201-DQB10303, HLA-DQA10301-DQB10301, HLA-DQA10501-DQB10301, HLA-DQA10501-DQB10302, HLA-DQA10501-DQB10303 | 0.8509 | Non-toxin | Allergen | 97.78% | Positive | Inducer |
| **CALIQITKRVPIFQD** | **275-289** | **DRB1_0103, DRB1_0801, DRB1_0802, DRB1_1101, DRB1_1301, DRB4_0103** | **0.4348** | **Non-toxin** | **Non-allergen** | **94.44%** | **Positive** | **Inducer** |
| FEEVVQTLASLATVV | 83-97 | DRB1_0404, DRB1_0901, HLA-DQA10102-DQB10501, HLA-DQA10103-DQB10603, HLA-DQA10201-DQB10202, HLA-DQA10201-DQB10301, HLA-DQA10201-DQB10303, HLA-DQA10501-DQB10303 | 0.2181 | Non-toxin | Non-allergen | 90.00% | Positive | Inducer |
| SFEEVVQTLASLATV | 82-96 | DRB1_0901, HLA-DQA10102-DQB10501, HLA-DQA10103-DQB10603, HLA-DQA10201-DQB10202, HLA-DQA10201-DQB10301, HLA-DQA10201-DQB10303, HLA-DQA10501-DQB10303 | 0.3477 | Non-toxin | Non-allergen | 90.00% | Positive | Inducer |
| VMTTGRATATAAATE | 146-160 | HLA-DQA10201-DQB10301, HLA-DQA10201-DQB10303, HLA-DQA10501-DQB10301, HLA-DQA10501-DQB10302, HLA-DQA10501-DQB10303 | 0.7806 | Non-toxin | Non-allergen | 96.67% | Positive | Non-inducer |
| ATATAAATEAYWAEH | 152-166 | HLA-DQA10102-DQB10602, HLA-DQA10201-DQB10301, HLA-DQA10201-DQB10303, HLA-DQA10501-DQB10301, HLA-DQA10501-DQB10302 | 0.5946 | Non-toxin | Allergen | 96.67% | Positive | Non-inducer |
| LVMTTGRATATAAAT | 145-159 | HLA-DQA10201-DQB10301, HLA-DQA10201-DQB10303, HLA-DQA10201-DQB10402, HLA-DQA10501-DQB10301, HLA-DQA10501-DQB10302, HLA-DQA10501-DQB10303, HLA-DQA10501-DQB10402 | 0.8489 | Non-toxin | Allergen | 96.67% | Positive | Non-inducer |
| RATATAAATEAYWAE | 151-165 | HLA-DQA10102-DQB10602, HLA-DQA10201-DQB10301, HLA-DQA10201-DQB10303, HLA-DQA10501-DQB10301, HLA-DQA10501-DQB10302, HLA-DQA10501-DQB10303 | 0.7306 | Non-toxin | Allergen | 96.67% | Positive | Non-inducer |
| DLLVMTTGRATATAA | 143-157 | DRB1_0801, DRB4_0103, HLA-DQA10102-DQB10501, HLA-DQA10201-DQB10301, HLA-DQA10201-DQB10303, HLA-DQA10201-DQB10402, HLA-DQA10501-DQB10301, HLA-DQA10501-DQB10303, HLA-DQA10501-DQB10402 | 0.7329 | Non-toxin | Non-allergen | 94.44% | Positive | Non-inducer |
| EEVVQTLASLATVVQ | 84-98 | DRB1_0404, DRB1_0901, HLA-DQA10102-DQB10501, HLA-DQA10103-DQB10603, HLA-DQA10201-DQB10301, HLA-DQA10201-DQB10303, HLA-DQA10501-DQB10303 | 0.222 | Non-toxin | Non-allergen | 92.22% | Positive | Non-inducer |
| EVVQTLASLATVVQQ | 85-99 | DRB1_0404, HLA-DQA10102-DQB10501, HLA-DQA10103-DQB10603, HLA-DQA10201-DQB10301, HLA-DQA10201-DQB10303, HLA-DQA10501-DQB10303 | 0.359 | Non-toxin | Non-allergen | 93.33% | Positive | Non-inducer |
| KYDLLVMTTGRATAT | 141-155 | DRB1_0404, DRB1_0801, DRB4_0103, HLA-DQA10102-DQB10501, HLA-DQA10201-DQB10301, HLA-DQA10201-DQB10303 | 0.8037 | Non-toxin | Non-allergen | 95.56% | Positive | Non-inducer |
| LLVMTTGRATATAAA | 144-158 | DRB4_0103, HLA-DQA10102-DQB10501, HLA-DQA10201-DQB10301, HLA-DQA10201-DQB10303, HLA-DQA10201-DQB10402, HLA-DQA10501-DQB10301, HLA-DQA10501-DQB10302, HLA-DQA10501-DQB10303, HLA-DQA10501-DQB10402 | 0.7727 | Non-toxin | Non-allergen | 94.44% | Positive | Non-inducer |
| VQTLASLATVVQQQT | 87-101 | HLA-DQA10102-DQB10501, HLA-DQA10102-DQB10602, HLA-DQA10103-DQB10603, HLA-DQA10201-DQB10301, HLA-DQA10201-DQB10303, HLA-DQA10501-DQB10303 | 0.5059 | Non-toxin | Non-allergen | 94.44% | Positive | Non-inducer |
| VVQTLASLATVVQQQ | 86-100 | HLA-DQA10102-DQB10501, HLA-DQA10102-DQB10602, HLA-DQA10103-DQB10603, HLA-DQA10201-DQB10301, HLA-DQA10201-DQB10303, HLA-DQA10501-DQB10303 | 0.5357 | Non-toxin | Non-allergen | 94.44% | Positive | Non-inducer |
| YDLLVMTTGRATATA | 142-156 | DRB1_0404, DRB1_0801, DRB4_0103, HLA-DQA10102-DQB10501, HLA-DQA10201-DQB10301, HLA-DQA10201-DQB10303, HLA-DQA10201-DQB10402, HLA-DQA10501-DQB10301, HLA-DQA10501-DQB10303 | 0.8252 | Non-toxin | Non-allergen | 94.44% | Positive | Non-inducer |

The selected epitopes have been shown in bold.

Table S8

Results of HTL epitope prediction of VP24 protein from EBOV

| **Epitope** | **Position** | **Allels** | **Antigenicity** | **Toxicity** | **Allergenicity** | **Conservancy** | **IFN-gamma** | **IL-4** |
| --- | --- | --- | --- | --- | --- | --- | --- | --- |
| NILKFINKLDALHVV | 156-170 | DRB1_0101, DRB1_0404, DRB1_0405, DRB1_0802, DRB1_1201, DRB4_0101, HLA-DPA10103-DPB10402 | 0.2840 | Non-toxin | Allergen | 72.55% | Positive | Inducer |
| TNTNHFNMRTQRVKE | 129-143 | DRB4_0103, HLA-DQA10201-DQB10402, HLA-DQA10303-DQB10402, HLA-DQA10501-DQB10402, HLA-DQA10601-DQB10402 | 0.5847 | Non-toxin | Allergen | 92.16% | Positive | Inducer |
| ILKFINKLDALHVVN | 157-171 | DRB1_0101, DRB1_0405, DRB1_0802, DRB1_1201, DRB4_0101, HLA-DPA10103-DPB10402 | 0.4893 | Non-toxin | Non-allergen | 72.55% | Positive | Inducer |
| MLSLIRSNILKFINK | 149-163 | DRB1_0103, DRB1_0701, DRB1_1301, DRB1_1302, DRB1_1501, DRB3_0301, HLA-DPA10201-DPB10501, HLA-DPA10301-DPB10402 | -0.3916 | Non-toxin | Non-allergen | 70.59% | Positive | Inducer |
| SLIRSNILKFINKLD | 151-165 | DRB1_0103, DRB1_1302, DRB3_0301, HLA-DPA10201-DPB10501, HLA-DPA10301-DPB10402 | -0.5384 | Non-toxin | Allergen | 70.59% | Positive | Inducer |
| LSLKMLSLIRSNILK | 145-159 | DRB1_0101, DRB1_0103, DRB1_0401, DRB1_0404, DRB1_0701, DRB1_0802, DRB1_1001, DRB1_1201, DRB1_1301, DRB1_1501, DRB1_1602, DRB4_0101, DRB4_0103, HLA-DPA10201-DPB10501, HLA-DPA10301-DPB10402, HLA-DQA10102-DQB10501 | 0.2413 | Non-toxin | Allergen | 94.12% | Positive | Non-inducer |
| QLSLKMLSLIRSNIL | 144-158 | DRB1_0404, DRB1_0701, DRB1_0802, DRB1_1001, DRB1_1201, DRB1_1301, DRB1_1501, DRB1_1602, DRB4_0101, HLA-DQA10102-DQB10501, HLA-DQA10102-DQB10602 | 0.7832 | Non-toxin | Allergen | 94.12% | Positive | Non-inducer |
| **EQLSLKMLSLIRSNI** | **143-157** | **DRB1_0404, DRB1_0802, DRB1_1001, DRB1_1201, DRB4_0101** | **0.7149** | **Non-toxin** | **Non-allergen** | **94.12%** | **Positive** | **Inducer** |
| KMLSLIRSNILKFIN | 148-162 | DRB1_0101, DRB1_0103, DRB1_0401, DRB1_0404, DRB1_0701, DRB1_1201, DRB1_1301, DRB1_1302, DRB1_1501, DRB3_0301, DRB4_0101, HLA-DPA10201-DPB10501, HLA-DPA10301-DPB10402 | -0.3656 | Non-toxin | Non-allergen | 96.08% | Positive | Inducer |
| LKFINKLDALHVVNY | 158-172 | DRB1_0101, DRB1_0405, DRB1_0802, DRB1_1201, DRB4_0101 | 0.9515 | Non-toxin | Non-allergen | 72.55% | Positive | Inducer |
| **NHFNMRTQRVKEQLS** | **132-146** | **DRB1_0801, DRB4_0103, HLA-DQA10201-DQB10402, HLA-DQA10303-DQB10402, HLA-DQA10501-DQB10402, HLA-DQA10601-DQB10402** | **0.5202** | **Non-toxin** | **Non-allergen** | **92.16%** | **Positive** | **Inducer** |
| **NTNHFNMRTQRVKEQ** | **130-144** | **DRB1_0801, DRB4_0103, HLA-DQA10201-DQB10402, HLA-DQA10303-DQB10402, HLA-DQA10501-DQB10402, HLA-DQA10601-DQB10402** | **0.6166** | **Non-toxin** | **Non-allergen** | **92.16%** | **Positive** | **Inducer** |
| SNILKFINKLDALHV | 155-169 | DRB1_0101, DRB1_0405, DRB1_0802, DRB1_1201, DRB1_1501, DRB4_0101 | 0.1688 | Non-toxin | Non-allergen | 72.55% | Positive | Inducer |
| **TNHFNMRTQRVKEQL** | **131-145** | **DRB1_0801, DRB4_0103, HLA-DQA10201-DQB10402, HLA-DQA10303-DQB10402, HLA-DQA10501-DQB10402, HLA-DQA10601-DQB10402** | **0.4209** | **Non-toxin** | **Non-allergen** | **92.16%** | **Positive** | **Inducer** |
| LKMLSLIRSNILKFI | 147-161 | DRB1_0101, DRB1_0103, DRB1_0401, DRB1_0404, DRB1_0701, DRB1_1201, DRB1_1301, DRB1_1302 DRB1_1501, DRB1_1602, DRB3_0301, DRB4_0101, DRB4_0103, HLA-DPA10103-DPB10401, HLA-DPA10201-DPB10501, HLA-DPA10301-DPB10402, HLA-DQA10102-DQB10501 | -0.3557 | Non-toxin | Non-allergen | 94.12% | Positive | Non-inducer |
| LSLIRSNILKFINKL | 150-164 | DRB1_0103, DRB1_0701, DRB1_1301, DRB1_1302, DRB1_1501, DRB3_0301, HLA-DPA10201-DPB10501, HLA-DPA10301-DPB10402 | -0.506 | Non-toxin | Non-allergen | 70.59% | Positive | Non-inducer |
| SLKMLSLIRSNILKF | 146-160 | DRB1_0101, DRB1_0103, DRB1_0401, DRB1_0404, DRB1_0405, DRB1_0701, DRB1_0802, DRB1_1001, DRB1_1201, DRB1_1301, DRB1_1302, DRB1_1501, DRB1_1602, DRB3_0301, DRB4_0101, DRB4_0103, HLA-DPA10201-DPB10501, HLA-DPA10301-DPB10402, HLA-DQA10102-DQB10501 | -0.0764 | Non-toxin | Non-allergen | 94.12% | Positive | Non-inducer |

The selected epitopes have been shown in bold

Table S9

Results of HTL epitope prediction of VP30 protein from EBOV

| **Epitope** | **Position** | **Allels** | **Antigenicity** | **Toxicity** | **Allergenicity** | **Conservancy** | **IFN-gamma** | **IL-4** |
| --- | --- | --- | --- | --- | --- | --- | --- | --- |
| ITLLTLIKTAEHWAR | 142-156 | DRB5_0101, DRB1_0404, DRB1_0405, DRB1_0801, DRB1_0802, HLA-DQA10102-DQB10501 | 0.3864 | Non-toxin | Non-allergen | 100.00% | Positive | Non-inducer |
| **LLTLCAVMTRKFSKS** | **170-184** | **DRB1_0801, DRB1_1101, DRB1_1301, DRB4_0103, HLA-DQA10601-DQB10402** | **0.4165** | **Non-toxin** | **Non-allergen** | **100.00%** | **Positive** | **Inducer** |
| LTLCAVMTRKFSKSQ | 171-185 | DRB1_0801, DRB1_1101, DRB1_1301, DRB4_0103, HLA-DQA10601-DQB10402 | 0.4852 | Non-toxin | Allergen | 100.00% | Positive | Inducer |

The selected epitopes have been shown in bold

Table S10

Results of HTL epitope prediction of VP40 protein from EBOV

| **Epitope** | **Position** | **Allels** | **Antigenicity** | **Toxicity** | **Allergenicity** | **Conservancy** | **IFN-gamma** | **IL-4** |
| --- | --- | --- | --- | --- | --- | --- | --- | --- |
| EAIYPVRSNSTIARG | 15-29 | DRB1_0401, DRB1_0404, DRB1_0802, HLA-DQA10102-DQB10501, HLA-DQA10501-DQB10402 | 0.4078 | Non-toxin | Allergen | 23.68% | Positive | Inducer |
| EYMEAIYPVRSNSTI | 12-26 | DRB1_0404, HLA-DQA10201-DQB10402, HLA-DQA10303-DQB10402, HLA-DQA10501-DQB10402, HLA-DQA10601-DQB10402 | 0.5735 | Non-toxin | Allergen | 27.63% | Positive | Inducer |
| MEAIYPVRSNSTIAR | 14-28 | DRB1_0401, DRB1_0404, DRB1_0802, HLA-DQA10102-DQB10501, HLA-DQA10201-DQB10402, HLA-DQA10501-DQB10402, HLA-DQA10601-DQB10402 | 0.4276 | Non-toxin | Allergen | 27.63% | Positive | Inducer |
| QAFLQEFVLPPVQLP | 155-169 | HLA-DPA10103-DPB10401, HLA-DPA10201-DPB10101, HLA-DQA10101-DQB10501, HLA-DQA10102-DQB10502, HLA-DQA10104-DQB10503 | -0.1497 | Non-toxin | Allergen | 92.11% | Positive | Inducer |
| **STTAAIMLASYTITH** | **110-124** | **DRB1_1501, HLA-DQA10102-DQB10602, HLA-DQA10201-DQB10301, HLA-DQA10201-DQB10303, HLA-DQA10501-DQB10301, HLA-DQA10501-DQB10303** | **0.6395** | **Non-toxin** | **Non-allergen** | **96.05%** | **Positive** | **Inducer** |
| YMEAIYPVRSNSTIA | 13-27 | DRB1_0401, DRB1_0404, DRB1_0802, HLA-DQA10201-DQB10402, HLA-DQA10303-DQB10402, HLA-DQA10501-DQB10402, HLA-DQA10601-DQB10402 | 0.6527 | Non-toxin | Allergen | 27.63% | Positive | Inducer |
| DSTTAAIMLASYTIT | 109-123 | HLA-DQA10102-DQB10602, HLA-DQA10201-DQB10301, HLA-DQA10201-DQB10303, HLA-DQA10501-DQB10301, HLA-DQA10501-DQB10303 | 0.547 | Non-toxin | Non-allergen | 96.05% | Positive | Non-inducer |
| FDSTTAAIMLASYTI | 108-122 | HLA-DQA10102-DQB10602, HLA-DQA10201-DQB10301, HLA-DQA10201-DQB10303, HLA-DQA10501-DQB10301, HLA-DQA10501-DQB10303 | 0.5316 | Non-toxin | Non-allergen | 96.05% | Positive | Non-inducer |
| KTYSFDSTTAAIMLA | 104-118 | DRB1_0401, DRB3_0101, HLA-DQA10201-DQB10202, HLA-DQA10201-DQB10301, HLA-DQA10201-DQB10303, HLA-DQA10201-DQB10402, HLA-DQA10501-DQB10302, HLA-DQA10501-DQB10303 | 0.7349 | Non-toxin | Non-allergen | 93.42% | Positive | Non-inducer |
| QKTYSFDSTTAAIML | 103-117 | DRB1_0401, DRB1_0701, DRB3_0101, HLA-DQA10201-DQB10202, HLA-DQA10201-DQB10301, HLA-DQA10201-DQB10303, HLA-DQA10201-DQB10402, HLA-DQA10501-DQB10302, HLA-DQA10501-DQB10303 | 0.7565 | Non-toxin | Non-allergen | 93.42% | Positive | Non-inducer |
| SFDSTTAAIMLASYT | 107-121 | HLA-DQA10102-DQB10602, HLA-DQA10201-DQB10301, HLA-DQA10201-DQB10303, HLA-DQA10501-DQB10301, HLA-DQA10501-DQB10302, HLA-DQA10501-DQB10303 | 0.6233 | Non-toxin | Non-allergen | 96.05% | Positive | Non-inducer |
| TYSFDSTTAAIMLAS | 105-119 | DRB1_0401, DRB3_0101, HLA-DQA10102-DQB10602, HLA-DQA10201-DQB10202, HLA-DQA10201-DQB10301, HLA-DQA10201-DQB10303, HLA-DQA10201-DQB10402, HLA-DQA10501-DQB10302, HLA-DQA10501-DQB10303 | 0.5351 | Non-toxin | Non-allergen | 93.42% | Positive | Non-inducer |
| YSFDSTTAAIMLASY | 106-120 | HLA-DQA10102-DQB10602, HLA-DQA10201-DQB10301, HLA-DQA10201-DQB10303, HLA-DQA10501-DQB10301, HLA-DQA10501-DQB10302, HLA-DQA10501-DQB10303 | 0.6272 | Non-toxin | Non-allergen | 93.42% | Positive | Non-inducer |

The selected epitopes have been shown in bold.

Table S11

Results of HTL epitope prediction of GP protein from EBOV

| **Epitope** | **Position** | **Allels** | **Antigenicity** | **Toxicity** | **Allergenicity** | **Conservancy** | **IFN-gamma** | **IL-4** |
| --- | --- | --- | --- | --- | --- | --- | --- | --- |
| AAVSHLTTLATISTS | 360-374 | DRB1_0404, HLA-DQA10102-DQB10501, HLA-DQA10103-DQB10603, HLA-DQA10201-DQB10301, HLA-DQA10201-DQB10303, HLA-DQA10201-DQB10402, HLA-DQA10501-DQB10303 | 0.5253 | Non-toxin | Non-allergen | 45.24% | Positive | Inducer |
| AVSHLTTLATISTSP | 361-375 | DRB1_0404, HLA-DQA10102-DQB10501, HLA-DQA10103-DQB10603, HLA-DQA10201-DQB10301, HLA-DQA10201-DQB10303, HLA-DQA10201-DQB10402, HLA-DQA10501-DQB10303 | 0.5814 | Non-toxin | Non-allergen | 41.67% | Positive | Inducer |
| **DRFKRTSFFLWVIIL** | **12-26** | **HLA-DPA10103-DPB10401, HLA-DPA10201-DPB10101, HLA-DPA10201-DPB10501, HLA-DPA10201-DPB11401, HLA-DPA10103-DPB10201** | **0.5169** | **Non-toxin** | **Non-allergen** | **97.62%** | **Positive** | **Inducer** |
| EAAVSHLTTLATIST | 359-373 | DRB1_0404, HLA-DQA10102-DQB10501, HLA-DQA10103-DQB10603, HLA-DQA10201-DQB10301, HLA-DQA10201-DQB10303, HLA-DQA10501-DQB10303 | 0.4448 | Non-toxin | Allergen | 23.81% | Positive | Inducer |
| **EYLFEVDNLTYVQLE** | **231-245** | **DRB1_0401, DRB3_0101, HLA-DPA10301-DPB10402, HLA-DQA10301-DQB10302, HLA-DQA10401-DQB10402** | **0.7918** | **Non-toxin** | **Non-allergen** | **100.00%** | **Positive** | **Inducer** |
| HLTTLATISTSPQSL | 364-378 | DRB1_0405, DRB1_0802, HLA-DQA10102-DQB10501, HLA-DQA10103-DQB10603, HLA-DQA10201-DQB10301, HLA-DQA10201-DQB10303, HLA-DQA10201-DQB10402, HLA-DQA10501-DQB10303 | 0.6908 | Non-toxin | Non-allergen | 7.14% | Positive | Inducer |
| **ILFQRTFSIPLGVIH** | **25-39** | **DRB1_0101, DRB1_0701, DRB1_1602, HLA-DPA10103-DPB10301, HLA-DPA10201-DPB11401** | **0.6845** | **Non-toxin** | **Non-allergen** | **98.81%** | **Positive** | **Inducer** |
| **LFEVDNLTYVQLESR** | **233-247** | **DRB3_0101, HLA-DPA10201-DPB10101, HLA-DPA10301-DPB10402, HLA-DQA10301-DQB10302, HLA-DQA10401-DQB10402** | **1.1033** | **Non-toxin** | **Non-allergen** | **97.62%** | **Positive** | **Inducer** |
| LPRDRFKRTSFFLWV | 9-23 | HLA-DPA10103-DPB10401, HLA-DPA10201-DPB10101, HLA-DPA10201-DPB10501, HLA-DPA10201-DPB11401, HLA-DPA10103-DPB10201 | 0.0313 | Non-toxin | Non-allergen | 97.62% | Positive | Inducer |
| LTTLATISTSPQSLT | 365-379 | DRB1_0405, HLA-DQA10102-DQB10501, HLA-DQA10103-DQB10603, HLA-DQA10201-DQB10301, HLA-DQA10201-DQB10303, HLA-DQA10201-DQB10402, HLA-DQA10501-DQB10303 | 0.7035 | Non-toxin | Non-allergen | 7.14% | Positive | Inducer |
| **NETEYLFEVDNLTYV** | **228-242** | **DRB1_0401, DRB3_0101, HLA-DQA10101-DQB10501, HLA-DQA10201-DQB10202, HLA-DQA10501-DQB10201** | **0.6229** | **Non-toxin** | **Non-allergen** | **100.00%** | **Positive** | **Inducer** |
| PRDRFKRTSFFLWVI | 10-24 | HLA-DPA10103-DPB10401, HLA-DPA10201-DPB10101, HLA-DPA10201-DPB10501, HLA-DPA10201-DPB11401, HLA-DPA10103-DPB10201 | 0.35 | Non-toxin | Allergen | 97.62% | Positive | Inducer |
| **RDRFKRTSFFLWVII** | **11-25** | **HLA-DPA10103-DPB10401, HLA-DPA10201-DPB10101, HLA-DPA10201-DPB10501, HLA-DPA10201-DPB11401, HLA-DPA10103-DPB10201** | **0.4177** | **Non-toxin** | **Non-allergen** | **97.62%** | **Positive** | **Inducer** |
| **RFKRTSFFLWVIILF** | **13-27** | **HLA-DPA10103-DPB10401, HLA-DPA10103-DPB10601, HLA-DPA10201-DPB10101, HLA-DPA10201-DPB10501, HLA-DPA10201-DPB11401, HLA-DPA10103-DPB10201** | **0.8986** | **Non-toxin** | **Non-allergen** | **97.62%** | **Positive** | **Inducer** |
| SHLTTLATISTSPQS | 363-377 | DRB1_0405, DRB1_0802, HLA-DQA10102-DQB10501, HLA-DQA10103-DQB10603, HLA-DQA10201-DQB10301, HLA-DQA10201-DQB10303, HLA-DQA10201-DQB10402, HLA-DQA10501-DQB10303 | 0.5747 | Non-toxin | Non-allergen | 7.14% | Positive | Inducer |
| TTLATISTSPQSLTT | 366-380 | HLA-DQA10102-DQB10501, HLA-DQA10103-DQB10603, HLA-DQA10201-DQB10301, HLA-DQA10201-DQB10303, HLA-DQA10201-DQB10402, HLA-DQA10501-DQB10303 | 0.6511 | Non-toxin | Non-allergen | 7.14% | Positive | Inducer |
| VSHLTTLATISTSPQ | 362-376 | HLA-DQA10102-DQB10501, HLA-DQA10103-DQB10603, HLA-DQA10201-DQB10301, HLA-DQA10201-DQB10303, HLA-DQA10201-DQB10402, HLA-DQA10501-DQB10303 | 0.6416 | Non-toxin | Allergen | 41.67% | Positive | Inducer |
| EELSFTVVSNGAKNI | 304-318 | DRB1_0403, DRB1_1302, DRB3_0301, DRB5_0101, HLA-DQA10201-DQB10402 | 0.9258 | Non-toxin | Non-allergen | 5.95% | Positive | Non-inducer |
| ELSFTVVSNGAKNIS | 305-319 | DRB1_0403, DRB1_1302, DRB3_0301, DRB5_0101, HLA-DQA10201-DQB10402 | 1.1108 | Non-toxin | Non-allergen | 5.95% | Positive | Non-inducer |
| FFLYDRLASTVIYRG | 159-173 | DRB3_0101, HLA-DPA10103-DPB10301, HLA-DPA10201-DPB10101, HLA-DPA10301-DPB10402, HLA-DQA10102-DQB10602 | 0.401 | Non-toxin | Non-allergen | 98.81% | Positive | Non-inducer |
| GKLGLITNTIAGVAG | 477-491 | DRB1_0403, DRB1_1302, DRB3_0301, HLA-DQA10102-DQB10501, HLA-DQA10103-DQB10603, HLA-DQA10201-DQB10303 | 0.8965 | Non-toxin | Non-allergen | 47.62% | Positive | Non-inducer |
| GLITNTIAGVAGLIT | 480-494 | DRB1_0403, DRB3_0301, HLA-DQA10102-DQB10501, HLA-DQA10103-DQB10603, HLA-DQA10201-DQB10301, HLA-DQA10201-DQB10303, HLA-DQA10501-DQB10303 | 0.4402 | Non-toxin | Non-allergen | 94.05% | Positive | Non-inducer |
| IILFQRTFSIPLGVI | 24-38 | DRB1_0101, DRB1_0701, DRB1_1602, HLA-DPA10103-DPB10301, HLA-DPA10201-DPB11401 | 0.6978 | Non-toxin | Non-allergen | 98.81% | Positive | Non-inducer |
| KLGLITNTIAGVAGL | 478-492 | DRB1_0403, DRB1_1302, DRB3_0301, HLA-DQA10102-DQB10501, HLA-DQA10103-DQB10603, HLA-DQA10201-DQB10303, HLA-DQA10501-DQB10303 | 0.8654 | Non-toxin | Non-allergen | 47.62% | Positive | Non-inducer |
| LGLITNTIAGVAGLI | 479-493 | DRB1_0403, DRB1_1302, DRB3_0301, HLA-DQA10102-DQB10501, HLA-DQA10103-DQB10603, HLA-DQA10201-DQB10301, HLA-DQA10201-DQB10303, HLA-DQA10501-DQB10303 | 0.6367 | Non-toxin | Non-allergen | 94.05% | Positive | Non-inducer |
| SGKLGLITNTIAGVA | 476-490 | DRB1_0403, DRB1_1302, DRB3_0301, HLA-DQA10102-DQB10501, HLA-DQA10103-DQB10603 | 0.8295 | Non-toxin | Non-allergen | 47.62% | Positive | Non-inducer |
| TELRTFSILNRKAID | 577-591 | DRB1_0801, DRB1_1101, HLA-DPA10103-DPB10301, HLA-DPA10201-DPB10501, HLA-DQA10201-DQB10402 | 0.9403 | Non-toxin | Allergen | 97.62% | Positive | Non-inducer |
| TTELRTFSILNRKAI | 576-590 | DRB1_0801, DRB1_1101, HLA-DPA10103-DPB10301, HLA-DPA10201-DPB10501, HLA-DQA10201-DQB10402 | 0.8978 | Non-toxin | Allergen | 97.62% | Positive | Non-inducer |
| VIILFQRTFSIPLGV | 23-37 | DRB1_0101, DRB1_0401, DRB1_0701, DRB1_1501, HLA-DPA10103-DPB10301, HLA-DPA10201-DPB11401 | 0.8174 | Non-toxin | Non-allergen | 98.81% | Positive | Non-inducer |

The selected epitopes have been shown in bold.

Table S12

Results of HTL epitope prediction of NP protein from EBOV

| **Epitope** | **Position** | **Allels** | **Antigenicity** | **Toxicity** | **Allergenicity** | **Conservancy** | **IFN-gamma** | **IL-4** |
| --- | --- | --- | --- | --- | --- | --- | --- | --- |
| NIKRTLAAMPEEETT | 129-143 | HLA-DPA10103-DPB10301, HLA-DQA10301-DQB10302, HLA-DQA10401-DQB10402, HLA-DQA10501-DQB10201, HLA-DQA10501-DQB10302 | 0.5268 | Non-toxin | Allergen | 98.92% | Positive | Inducer |
| NSFKAALSSLAKHGE | 278-292 | DRB 1_0101, DRB1_0401, DRB1_0405, DRB1_0802, DRB1_0901, DRB1_1001, DRB1_1602, DRB5_0101 | 0.0439 | Non-toxin | Allergen | 95.70% | Positive | Inducer |
| **QQGIVRQRVIPVYQV** | **32-46** | **DRB1_0103, DRB1_1302, DRB4_0101, DRB4_0103, HLA-DPA10103-DPB10301** | **0.5571** | **Non-toxin** | **Non-allergen** | **96.77%** | **Positive** | **Inducer** |
| **VQQGIVRQRVIPVYQ** | **31-45** | **DRB1_0103, DRB1_1302, DRB4_0101, DRB4_0103, HLA-DPA10103-DPB10301** | **0.5424** | **Non-toxin** | **Non-allergen** | **96.77%** | **Positive** | **Inducer** |
| AGQFLSFASLFLPKL | 147-161 | DRB1_0402, DRB1_1001, DRB1_1602, HLA-DPA10103-DPB10401, HLA-DPA10201-DPB10101, HLA-DPA10301-DPB10402, HLA-DPA10103-DPB10201 | 0.2007 | Non-toxin | Allergen | 100.00% | Positive | Inducer |
| EVNSFKAALSSLAKH | 276-290 | DRB1_0101, DRB1_0401, DRB1_0405, DRB1_0802, DRB1_0901, DRB1_1001, DRB1_1602, DRB5_0101, HLA-DQA10201-DQB10402, HLA-DQA10303-DQB10402, HLA-DQA10501-DQB10301, HLA-DQA10501-DQB10402 | -0.0987 | Non-toxin | Allergen | 95.70% | Positive | Inducer |
| IKFLLIHQGMHMVAG | 210-224 | DRB1_0101, DRB1_0402, DRB1_1301, DRB3_0301, DRB4_0101, HLA-DPA10103-DPB10402 | 0.7206 | Non-toxin | Allergen | 98.92% | Positive | Inducer |
| KFLLIHQGMHMVAGH | 211-225 | DRB1_0402, DRB1_1301, DRB4_0101, HLA-DPA10103-DPB10402, HLA-DQA10102-DQB10602 | 0.4675 | Non-toxin | Allergen | 98.92% | Positive | Inducer |
| KLTEAITAASLPKTS | 404-418 | HLA-DPA10201-DPB11401, HLA-DQA10201-DQB10301, HLA-DQA10201-DQB10303, HLA-DQA10501-DQB10301, HLA-DQA10501-DQB10303 | 0.3948 | Non-toxin | Allergen | 95.70% | Positive | Inducer |
| KNIKRTLAAMPEEET | 128-142 | HLA-DPA10103-DPB10301, HLA-DQA10301-DQB10302, HLA-DQA10401-DQB10402, HLA-DQA10501-DQB10201, HLA-DQA10501-DQB10302 | 0.3523 | Non-toxin | Allergen | 54.84% | Positive | Inducer |
| LIKFLLIHQGMHMVA | 209-223 | DRB1_0402, DRB1_0404, DRB1_1301, DRB3_0301, DRB4_0101, HLA-DPA10103-DPB10402, HLA-DPA10301-DPB10402 | 0.2917 | Non-toxin | Allergen | 98.92% | Positive | Inducer |
| NEVNSFKAALSSLAK | 275-289 | DRB1_0101, DRB1_0401, DRB1_0405, DRB1_0802, DRB1_0901, DRB1_1001, DRB1_1602, DRB5_0101, HLA-DQA10201-DQB10402, HLA-DQA10303-DQB10402, HLA-DQA10501-DQB10301, HLA-DQA10501-DQB10402 | -0.086 | Non-toxin | Allergen | 95.70% | Positive | Inducer |
| QGIVRQRVIPVYQVN | 33-47 | DRB1_0103, DRB1_1302, DRB4_0101, DRB4_0103, HLA-DPA10103-DPB10301 | 0.7058 | Non-toxin | Allergen | 96.77% | Positive | Inducer |
| RLAKLTEAITAASLP | 401-415 | HLA-DPA10201-DPB11401, HLA-DQA10201-DQB10301, HLA-DQA10201-DQB10303, HLA-DQA10501-DQB10302, HLA-DQA10501-DQB10303 | 0.305 | Non-toxin | Allergen | 95.70% | Positive | Inducer |
| SFKAALSSLAKHGEY | 279-293 | DRB1_0101, DRB1_0401, DRB1_0405, DRB1_0901, DRB1_1001, DRB5_0101 | 0.4828 | Non-toxin | Allergen | 95.70% | Positive | Inducer |
| VNSFKAALSSLAKHG | 277-291 | DRB1_0101, DRB1_0401, DRB1_0405, DRB1_0802, DRB1_0901, DRB1_1001, DRB1_1602, DRB5_0101 HLA-DQA10201-DQB10402, HLA-DQA10501-DQB10402 | -0.1259 | Non-toxin | Allergen | 95.70% | Positive | Inducer |
| **IKRTLAAMPEEETTE** | **130-144** | **HLA-DPA10103-DPB10301, HLA-DQA10301-DQB10302, HLA-DQA10401-DQB10402, HLA-DQA10501-DQB10201, HLA-DQA10501-DQB10302** | **0.4031** | **Non-toxin** | **Non-allergen** | **98.92%** | **Positive** | **Inducer** |
| MVIFRLMRTNFLIKF | 198-212 | DRB1_0101, DRB1_0103, DRB1_0401, DRB1_0405, DRB1_0701, DRB1_0801, DRB1_1001, DRB1_1201, DRB1_1301, DRB1_1501, DRB1_1602, DRB4_0103, DRB5_0101, HLA-DPA10103-DPB10401, HLA-DPA10201-DPB10501, HLA-DPA10301-DPB10402, HLA-DQA10501-DQB10402 | 0.3032 | Non-toxin | Non-allergen | 100.00% | Positive | Inducer |
| VIFRLMRTNFLIKFL | 199-213 | DRB1_0101, DRB1_0103, DRB1_0401, DRB1_0405, DRB1_0701, DRB1_0801, DRB1_1001, DRB1_1201, DRB1_1301, DRB1_1501, DRB4_0103, DRB5_0101, HLA-DPA10103-DPB10401, HLA-DPA10201-DPB10501, HLA-DPA10301-DPB10402, HLA-DQA10501-DQB10402 | 0.1598 | Non-toxin | Non-allergen | 98.92% | Positive | Inducer |
| GHMMVIFRLMRTNFL | 195-209 | DRB1_0101, DRB1_0103, DRB1_0401, DRB1_0701, DRB1_0801, DRB1_1201, DRB1_1301, DRB4_0103, HLA-DQA10501-DQB10402, HLA-DQA10601-DQB10402 | 0.1399 | Non-toxin | Allergen | 100.00% | Positive | Non-inducer |
| ATAHGSTLAGVNVGE | 324-338 | HLA-DQA10102-DQB10602, HLA-DQA10201-DQB10303, HLA-DQA10301-DQB10301, HLA-DQA10501-DQB10302, HLA-DQA10501-DQB10303 | 0.5661 | Non-toxin | Non-allergen | 100.00% | Positive | Non-inducer |
| HMMVIFRLMRTNFLI | 196-210 | DRB1_0101, DRB1_0103, DRB1_0401, DRB1_0405, DRB1_0701, DRB1_0801, DRB1_1001, DRB1_1201, DRB1_1301, DRB1_1501, DRB4_0103, DRB5_0101, HLA-DPA10301-DPB10402, HLA-DQA10501 DQB10402, HLA-DQA10601-DQB10402 | 0.3639 | Non-toxin | Non-allergen | 100.00% | Positive | Non-inducer |
| IALGVATAHGSTLAG | 319-333 | HLA-DQA10201-DQB10301, HLA-DQA10201-DQB10303, HLA-DQA10301-DQB10301, HLA-DQA10501-DQB10301, HLA-DQA10501-DQB10303 | 0.6289 | Non-toxin | Non-allergen | 100.00% | Positive | Non-inducer |
| GQFLSFASLFLPKLV | 148-162 | DRB1_0402, DRB1_1602, HLA-DPA10103-DPB10401, HLA-DPA10201-DPB10101, HLA-DPA10301, DPB10402, HLA-DPA10103-DPB10201 | 0.2298 | Non-toxin | Allergen | 100.00% | Positive | Non-inducer |
| GVATAHGSTLAGVNV | 322-336 | HLA-DQA10102-DQB10602, HLA-DQA10201-DQB10301, HLA-DQA10201-DQB10303, HLA-DQA10301-DQB10301, HLA-DQA10501-DQB10301, HLA-DQA10501-DQB10303 | 0.5221 | Non-toxin | Allergen | 100.00% | Positive | Non-inducer |
| LGVATAHGSTLAGVN | 321-335 | HLA-DQA10102-DQB10602, HLA-DQA10201-DQB10301, HLA-DQA10201-DQB10303, HLA-DQA10301-DQB10301, HLA-DQA10501-DQB10301, HLA-DQA10501-DQB10303 | 0.6384 | Non-toxin | Allergen | 100.00% | Positive | Non-inducer |
| VATAHGSTLAGVNVG | 323-337 | HLA-DQA10102-DQB10602, HLA-DQA10201-DQB10303, HLA-DQA10301-DQB10301, HLA-DQA10501-DQB10302, HLA-DQA10501-DQB10303 | 0.6351 | Non-toxin | Allergen | 100.00% | Positive | Non-inducer |
| AIALGVATAHGSTLA | 318-332 | HLA-DQA10201-DQB10301, HLA-DQA10201-DQB10303, HLA-DQA10301-DQB10301, HLA-DQA10501-DQB10301, HLA-DQA10501-DQB10303 | 0.6738 | Non-toxin | Non-allergen | 100.00% | Positive | Non-inducer |
| ALGVATAHGSTLAGV | 320-334 | HLA-DQA10201-DQB10301, HLA-DQA10201-DQB10303, HLA-DQA10301-DQB10301, HLA-DQA10501-DQB10301, HLA-DQA10501-DQB10303 | 0.5671 | Non-toxin | Non-allergen | 100.00% | Positive | Non-inducer |
| FLSFASLFLPKLVVG | 150-164 | DRB1_0402, HLA-DPA10103-DPB10401, HLA-DPA10201-DPB10101, HLA-DPA10301-DPB10402, HLA-DPA10103-DPB10201 | 0.2592 | Non-toxin | Non-allergen | 100.00% | Positive | Non-inducer |
| GVRLHPLARTAKVKN | 261-275 | DRB1_1301, DRB4_0103, HLA-DPA10103-DPB10301, HLA-DQA10303-DQB10402, HLA-DQA10501-DQB10402 | 1.0105 | Non-toxin | Non-allergen | 100.00% | Positive | Non-inducer |
| IFRLMRTNFLIKFLL | 200-214 | DRB1_0101, DRB1_0103, DRB1_0401, DRB1_0701, DRB1_1001, DRB1_1301, DRB1_1501, DRB4_0103, DRB5_0101, HLA-DPA10103-DPB10401, HLA-DPA10201-DPB10501, HLA-DPA10301-DPB10402 | -0.1089 | Non-toxin | Non-allergen | 98.92% | Positive | Non-inducer |
| MMVIFRLMRTNFLIK | 197-211 | DRB1_0101, DRB1_0103, DRB1_0401, DRB1_0405, DRB1_0701, DRB1_0801, DRB1_1001, DRB1_1201, DRB1_1301, DRB1_1501, DRB4_0103, DRB5_0101, HLA-DPA10103-DPB10401, HLA-DPA10201-DPB10501, HLA-DPA10301-DPB10402, HLA-DQA10501-DQB10402 | 0.3533 | Non-toxin | Non-allergen | 100.00% | Positive | Non-inducer |
| NAGQFLSFASLFLPK | 146-160 | DRB1_0402, DRB1_1602, HLA-DPA10103-DPB10401, HLA-DPA10201-DPB10101, HLA-DPA10103-DPB10201 | 0.2503 | Non-toxin | Non-allergen | 100.00% | Positive | Non-inducer |
| PQLSAIALGVATAHG | 314-328 | HLA-DQA10201-DQB10301, HLA-DQA10201-DQB10303, HLA-DQA10301-DQB10301, HLA-DQA10501-DQB10301, HLA-DQA10501-DQB10303 | 0.9657 | Non-toxin | Non-allergen | 100.00% | Positive | Non-inducer |
| QFLSFASLFLPKLVV | 149-163 | DRB1_0402, DRB1_1602, HLA-DPA10103-DPB10401, HLA-DPA10201-DPB10101, HLA-DPA10301-DPB10402, HLA-DPA10103-DPB10201 | 0.2918 | Non-toxin | Non-allergen | 100.00% | Positive | Non-inducer |
| QLSAIALGVATAHGS | 315-329 | HLA-DQA10201-DQB10301, HLA-DQA10201-DQB10303, HLA-DQA10301-DQB10301, HLA-DQA10501-DQB10301, HLA-DQA10501-DQB10303 | 0.9302 | Non-toxin | Non-allergen | 100.00% | Positive | Non-inducer |
| RGVRLHPLARTAKVK | 260-274 | DRB1_1301, DRB4_0103, HLA-DPA10103-DPB10301, HLA-DQA10303-DQB10402, HLA-DQA10501-DQB10402 | 0.9572 | Non-toxin | Non-allergen | 98.92% | Positive | Non-inducer |
| VGHMMVIFRLMRTNF | 194-208 | DRB1_0103, DRB1_0801, DRB1_1201, DRB1_1301, HLA-DQA10303-DQB10402, HLA-DQA10501-DQB10402, HLA-DQA10601-DQB10402 | 0.2676 | Non-toxin | Non-allergen | 100.00% | Positive | Non-inducer |
| VRLHPLARTAKVKNE | 262-276 | DRB1_1301, DRB4_0103, HLA-DQA10303-DQB10402, HLA-DQA10501-DQB10402, HLA-DPA10103-DPB10301, HLA-DQA10103-DQB10603 | 1.0289 | Non-toxin | Non-allergen | 100.00% | Positive | Non-inducer |

The selected epitopes have been shown in bold.

Table S13

Results of linear B-cell epitope prediction of VP35 protein from EBOV

| **Epitope** | **Position** | **Length** | **Antigenicity** | **Toxicity** | **Allergenicity** | **Conservancy** |
| --- | --- | --- | --- | --- | --- | --- |
| **QQTIASESLEQRITSLEN** | **99-116** | **18** | **0.5583** | **Non-toxin** | **Non-allergen** | **95.56%** |
| REAFNNLNSTTSLTEENFGKPDISAK | 197-222 | 26 | 0.6981 | Non-toxin | Non-allergen | 3.33% |
| **RGDIPRACQKSLRPVPPSPKID** | **300-321** | **22** | **0.4144** | **Non-toxin** | **Non-allergen** | **95.56%** |

The selected epitopes have been shown in bold.

Table S14

Results of linear B-cell epitope prediction of VP24 protein from EBOV

| **Epitope** | **Position** | **Length** | **Antigenicity** | **Toxicity** | **Allergenicity** | **Conservancy** |
| --- | --- | --- | --- | --- | --- | --- |
| **KTNDFAPAWSM** | **61-71** | **11** | **1.2175** | **Non-toxin** | **Non-allergen** | **94.12%** |
| LCNFLVSQTIQGW | 26-38 | 13 | -0.1356 | Non-toxin | Allergen | 78.43% |
| DQLIDQSLIE | 104-113 | 10 | -0.1013 | Non-toxin | Allergen | 100.00% |
| TGRYNLISPKKDLEKGVV | 5-22 | 18 | 0.3766 | Non-toxin | Non-allergen | 78.43% |
| **TNHFNMRTQRVKEQ** | **131-144** | **14** | **0.5681** | **Non-toxin** | **Non-allergen** | **92.16%** |
| EPDKSAMNRMKPGPAK | 203-218 | 16 | -0.0527 | Non-toxin | Non-allergen | 11.76% |
| KAFTQGSSTRMQS | 228-240 | 13 | 0.2101 | Non-toxin | Non-allergen | 94.12% |

The selected epitopes have been shown in bold.

Table S15

Results of linear B-cell epitope prediction of VP30 protein from EBOV

| **Epitope** | **Position** | **Length** | **Antigenicity** | **Toxicity** | **Allergenicity** | **Conservancy** |
| --- | --- | --- | --- | --- | --- | --- |
| HWARQDIRTIEDS | 153-165 | 13 | 0.4897 | Non-toxin | Allergen | 92.31% |
| **PQSDNEEASTNPGTCSWSD** | **266-284** | **19** | **0.4279** | **Non-toxin** | **Non-allergen** | **84.62%** |

The selected epitopes have been shown in bold.

Table S16

Results of linear B-cell epitope prediction of VP40 protein from EBOV

| **Epitope** | **Position** | **Length** | **Antigenicity** | **Toxicity** | **Allergenicity** | **Conservancy** |
| --- | --- | --- | --- | --- | --- | --- |
| QAFLQEFVLPPVQLPQY | 155-171 | 17 | -0.1399 | Non-toxin | Allergen | 90.79% |
| LPAATWTDDTPTGSNGALRPGISFHP | 186-211 | 26 | 0.3732 | Non-toxin | Non-allergen | 93.42% |
| **LPNKSGKKGNSADLTSPE** | **218-235** | **18** | **0.9858** | **Non-toxin** | **Non-allergen** | **90.79%** |
| QDFKIVPIDP | 245-254 | 10 | 2.3748 | Non-toxin | Allergen | 96.05% |
| VHKLTGKKVTSKNG | 268-281 | 14 | -0.1218 | Non-toxin | Non-allergen | 77.63% |
| CDTCHSPASLPA | 311-322 | 12 | -0.0205 | Non-toxin | Non-allergen | 96.05% |

The selected epitopes have been shown in bold.

Table S17

Results of linear B-cell epitope prediction of GP protein from EBOV

| **Epitope** | **Position** | **Length** | **Antigenicity** | **Toxicity** | **Allergenicity** | **Conservancy** |
| --- | --- | --- | --- | --- | --- | --- |
| **TLQVSDVDKLVCRDKLSSTNQL** | **42-63** | **22** | **0.5271** | **Non-toxin** | **Non-allergen** | **95.45%** |

The selected epitopes have been shown in bold.

Table S18

Results of linear B-cell epitope prediction of NP protein from EBOV

| **Epitope** | **Position** | **Length** | **Antigenicity** | **Toxicity** | **Allergenicity** | **Conservancy** |
| --- | --- | --- | --- | --- | --- | --- |
| IWMAPSLTESDMD | 8-20 | 13 | 0.7682 | Non-toxin | Allergen | 6.45% |
| GDYKLFLESG | 85-94 | 10 | -0.1124 | Non-toxin | Allergen | 87.10% |
| VKYLEGHGFRFEVKKRDGVKR | 96-116 | 21 | -0.5377 | Non-toxin | Allergen | 66.67% |
| EELLPAVSSGKN | 118-129 | 12 | 0.6497 | Non-toxin | Allergen | 55.91% |
| QVHAEQGLIQYPTA | 177-190 | 14 | -0.0254 | Non-toxin | Non-allergen | 100.00% |
| TLAGVNVGEQYQQLREAA | 330-347 | 18 | 0.7691 | Non-toxin | Allergen | 98.92% |

The selected epitopes have been shown in bold.
